# Diagnostic Stewardship in Community-Acquired Pneumonia With Syndromic Molecular Testing: A Randomized Clinical Trial

**DOI:** 10.1001/jamanetworkopen.2024.0830

**Published:** 2024-03-06

**Authors:** Dagfinn L. Markussen, Sondre Serigstad, Christian Ritz, Siri T. Knoop, Marit H. Ebbesen, Daniel Faurholt-Jepsen, Lars Heggelund, Cornelis H. van Werkhoven, Tristan W. Clark, Rune O. Bjørneklett, Øyvind Kommedal, Elling Ulvestad, Harleen M. S. Grewal

**Affiliations:** 1 Department of Clinical Science, Bergen Integrated Diagnostic Stewardship Cluster, University of Bergen, Bergen, Norway; 2 Emergency Care Clinic, Haukeland University Hospital, Bergen, Norway; 3 Department of Clinical Medicine, University of Bergen, Bergen, Norway; 4 National Institute of Public Health, University of Southern Denmark, Copenhagen, Denmark; 5 Department of Microbiology, Haukeland University Hospital, Bergen, Norway; 6 Department of Internal Medicine, Vestre Viken Hospital Trust, Drammen, Norway; 7 Julius Center for Health Sciences and Primary Care, University Medical Center Utrecht, Utrecht, the Netherlands; 8 School of Clinical and Experimental Sciences, Faculty of Medicine, University of Southampton, Southampton, United Kingdom

## Abstract

**Importance:**

Lower respiratory tract (LRT) infections, including community-acquired pneumonia (CAP), are a leading cause of hospital admissions and mortality. Molecular tests have the potential to optimize treatment decisions and management of CAP, but limited evidence exists to support their routine use.

**Objective:**

To determine whether the judicious use of a syndromic polymerase chain reaction (PCR)-based panel for rapid testing of CAP in the emergency department (ED) leads to faster, more accurate microbiological test result–based treatment.

**Design, Setting, and Participants:**

This parallel-arm, single-blinded, single-center, randomized clinical superiority trial was conducted between September 25, 2020, and June 21, 2022, in the ED of Haukeland University Hospital, a large tertiary care hospital in Bergen, Norway. Adult patients who presented to the ED with suspected CAP were recruited. Participants were randomized 1:1 to either the intervention arm or standard-of-care arm. The primary outcomes were analyzed according to the intention-to-treat principle.

**Intervention:**

Patients randomized to the intervention arm received rapid syndromic PCR testing (BioFire FilmArray Pneumonia plus Panel; bioMérieux) of LRT samples and standard of care. Patients randomized to the standard-of-care arm received standard microbiological diagnostics alone.

**Main Outcomes and Measures:**

The 2 primary outcomes were the provision of pathogen-directed treatment based on a microbiological test result and the time to provision of pathogen-directed treatment (within 48 hours after randomization).

**Results:**

There were 374 patients (221 males [59.1%]; median (IQR) age, 72 [60-79] years) included in the trial, with 187 in each treatment arm. Analysis of primary outcomes showed that 66 patients (35.3%) in the intervention arm and 25 (13.4%) in the standard-of-care arm received pathogen-directed treatment, corresponding to a reduction in absolute risk of 21.9 (95% CI, 13.5-30.3) percentage points and an odds ratio for the intervention arm of 3.53 (95% CI, 2.13-6.02; *P* < .001). The median (IQR) time to provision of pathogen-directed treatment within 48 hours was 34.5 (31.6-37.3) hours in the intervention arm and 43.8 (42.0-45.6) hours in the standard-of-care arm (mean difference, −9.4 hours; 95% CI, −12.7 to −6.0 hours; *P* < .001). The corresponding hazard ratio for intervention compared with standard of care was 3.08 (95% CI, 1.95-4.89). Findings remained significant after adjustment for season.

**Conclusions and Relevance:**

Results of this randomized clinical trial indicated that routine deployment of PCR testing for LRT pathogens led to faster and more targeted microbial treatment for patients with suspected CAP. Rapid molecular testing could complement or replace selected standard, time-consuming, laboratory-based diagnostics.

**Trial Registration:**

ClinicalTrials.gov Identifier: NCT04660084

## Key Points

**Question:**

Does the judicious use of a syndromic polymerase chain reaction (PCR)-based panel for rapid testing of patients hospitalized with suspected community-acquired pneumonia (CAP) lead to faster, more accurate microbiological test result–based treatment?

**Findings:**

In this randomized clinical trial of 374 patients, molecular testing significantly increased the proportion of patients with suspected CAP who received pathogen-directed treatment and reduced the median time to pathogen-directed treatment by 9.4 hours compared with standard of care.

**Meaning:**

Findings from this trial showed that routine deployment of PCR testing for lower respiratory tract pathogens enables faster and more targeted microbial treatment for patients with suspected CAP, suggesting that this tool could replace selected standard, time-consuming, laboratory-based diagnostics.

## Teaser

This randomized clinical trial evaluates a rapid syndromic polymerase chain reaction–based diagnostic test for lower respiratory tract infections in patients presenting to the emergency department.

## Introduction

Lower respiratory tract (LRT) infections, including community-acquired pneumonia (CAP), are a leading cause of hospital admissions and mortality, with an estimated 489 million incidents and 2.5 million deaths annually worldwide.^1,2,3^ Despite their substantial value, microbiological diagnosis and targeted treatment are not received by most patients.^4^ Although culture-based methods are considered to be a standard approach in the bacteriological diagnosis of CAP, they are labor intensive, detect a pathogen in only 20% to 40% of patients, and are insufficient to influence early decisions on antimicrobial therapy.^4,5^

Rapid syndromic polymerase chain reaction (PCR)-based panels have improved pathogen detection, potentially facilitating pathogen-directed treatment, reducing unnecessary use of antibiotics, and shortening hospital length of stay (LOS).^6,7^ Although the potential benefits of rapid PCR panels in CAP are clear, limited evidence currently supports their routine use. A few trials have examined patients with respiratory tract infections using molecular point-of-care tests (mPOCTs) for a combination of viruses and atypical bacteria, which yielded modest and conflicting results on antibiotic use and LOS.^8,9,10,11^ A recent randomized clinical trial examined patients with pneumonia using a comprehensive mPOCT and found an increase in result-directed therapy and de-escalation of antibiotics in the mPOCT group.^12^ However, the study included a mixture of patients from intensive care units with hospital-acquired pneumonia, ventilator-associated pneumonia, and CAP; thus, the findings may not be specifically applicable to patients with CAP.

In this trial, we aimed to determine whether the judicious use of a syndromic PCR-based panel for rapid testing of CAP in the emergency department (ED) leads to faster, more accurate microbiological test result–based treatment. Findings from this trial can potentially inform future guidelines on the management of CAP.

## Methods

### Study Design and Participants

This pragmatic, parallel-arm, single-blinded, single-center, randomized clinical superiority trial was conducted in the ED of Haukeland University Hospital, a large tertiary care institution in Bergen, Norway, that serves as a local hospital for approximately 470 000 residents and a referral hospital for 1 million people. The Regional Committee for Medical and Health Research Ethics in Norway approved the study protocol^13^ (Supplement 1). Written informed consent was obtained from all patients or their legal guardian or close relatives. We followed the Consolidated Standards of Reporting Trials (CONSORT) reporting guideline.

Patients were recruited from September 25, 2020, to June 1, 2021, and again from August 15, 2021, to June 21, 2022. Patients were eligible for inclusion if they were 18 years or older; presented to the ED with suspected CAP; and met at least 2 of the following criteria: new or worsening cough, new or worsening expectoration, new or worsening dyspnoea, hemoptysis, pleuritic chest pain, radiological evidence of pneumonia, abnormalities on chest auscultation and/or percussion, or fever (≥38.0 °C). Patients were ineligible if they had cystic fibrosis, had severe bronchiectasis, were hospitalized within the past 14 days prior to admission, were under a palliative approach (ie, life expectancy of <2 weeks), or were not willing to provide an LRT sample.^13^

### Randomization and Masking

**Figure 1.**
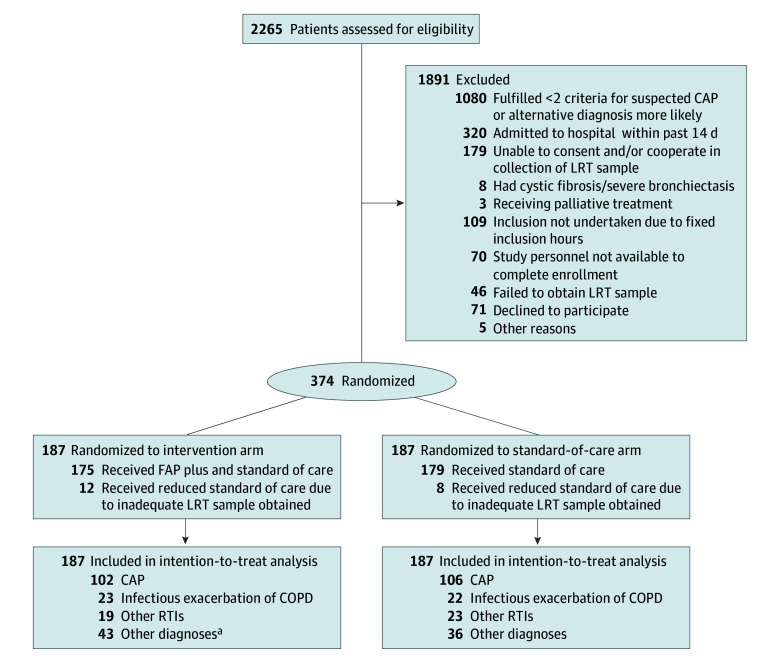
Flow Diagram of Study Participants CAP indicates community-acquired pneumonia; COPD, chronic obstructive pulmonary disease; FAP plus, BioFire FilmArray Pneumonia plus Panel; LRT, lower respiratory tract; RTI, respiratory tract infection. ^a^There was 1 withdrawal. One died within 48 hours after randomization.

Patients were randomized 1:1 to either the intervention arm (receiving rapid syndromic PCR testing in addition to standard-of-care microbiological diagnostics) or to the standard-of-care arm (receiving standard microbiological diagnostics alone) (Figure 1). Block randomization with varying block sizes (4, 6, or 8 patients per block) was applied using the R package blockrand (R Core Team). The allocation sequence was implemented in an electronic data capture system (Viedoc; Viedoc Technologies). Trial participants and ED clinicians were blinded to treatment group allocation.

### Procedures

Eligible patients were included shortly after presenting to the ED. Study nurses and physicians collected baseline information.

Rapid testing was performed using the BioFire FilmArray Pneumonia plus panel (FAP plus; bioMérieux). The FAP plus PCR test detects 27 bacterial and viral respiratory pathogens with 7 antimicrobial resistance genes, with the specific targets detailed in eTable 1 in Supplement 2. Standard-of-care methods included blood cultures, pneumococcal urine test (Sofia Streptococcus pneumoniae FIA; Quidel Corporation), and an in-house PCR test for oropharyngeal and/or nasopharyngeal swabs targeting respiratory viruses and atypical bacteria (ie, influenza A and B viruses, human parainfluenza viruses 1-3, respiratory syncytial virus, human metapneumovirus, rhinovirus, coronavirus [229E, OC43, HKU1, NL63], *Bordetella pertussis*, *Bordetella parapertussis*, *Mycoplasma pneumoniae,* and *Chlamydophilia pneumoniae*).

Results of the FAP plus PCR test, blood cultures, and urine tests for *Streptococcus pneumoniae* and *Legionella pneumophila* were communicated telephonically to the treating staff for both intervention and standard-of-care arms. The telephone call alerted staff that a test result was available in the patient’s electronic medical record. The report in the electronic medical record provided results, including standard responses suggesting whether the detected bacterium was a likely pathogen (eTable 2 in Supplement 2).

At admission, all patients were tested for SARS-CoV-2 infection using oropharyngeal or nasopharyngeal swabs tested on the GeneXpert system (Cepheid). The LRT samples were collected in the ED, primarily through sputum induction, using nebulized isotonic (0.9%) or hypertonic (5.8%) saline. Endotracheal aspiration was performed in case of an unsuccessful sputum induction and in patients with SARS-CoV-2 infection.

### Outcomes

Two primary outcomes were the (1) provision of pathogen-directed treatment based on a relevant microbiological test result and (2) time to provision of pathogen-directed treatment (within 48 hours after randomization). The first was a binary outcome, whereas the second was an event-time outcome wherein right censoring was present (ie, patients may cease participation due to death, discharge, or reaching 48 hours after randomization without receiving pathogen-directed treatment). Primary outcomes were defined for all patients who were randomized.

Two of us (D.L.M and S.S.) assessed whether and when a patient received pathogen-directed antimicrobial treatment based on a microbiological test result. In case of disagreement, a third physician (S.T.K.) arbitrated. To be considered as pathogen-directed treatment, documentation in the patient’s journal by the treating physician was required that described a change in antimicrobial treatment, continuation of an already correctly initiated antimicrobial treatment, or discontinuation of an antimicrobial treatment. The final diagnosis of CAP was established retrospectively through clinical adjudication using prespecified criteria (eTable 3 in Supplement 2).

Secondary outcomes included the binary outcomes: provision of any antibiotics, provision of narrow-spectrum antibiotics within 48 hours, provision of a single dose of antibiotics, antibiotics not used for more than 48 hours, treatment with intravenous antibiotics, de-escalation from broad-spectrum to narrow-spectrum antibiotics, and escalation from narrow-spectrum to broad-spectrum antibiotics. Continuous secondary outcomes included the duration of provision of antibiotics (days), intravenous antibiotics (days), broad-spectrum antibiotics (days), time from admission to the administration of antibiotics (hours), and turnaround time (hours; ie, time from admission to receiving a microbiological report (result of FAP plus PCR test and/or sputum culture). Broad-spectrum antibiotics were defined as penicillin with enzyme inhibitors, second- and third-generation cephalosporins, carbapenems, and quinolones.^14^ Additionally, LOS in days, mortality (30 days and 90 days), readmission within 30 days after discharge, and adverse outcomes were reported.

### Statistical Analysis

Because 2 primary outcomes were used, separate sample size calculations were performed for each outcome at a 2-sided significance level of .05/2 = .025 (instead of .05), assuming a power of 80%. To detect an increase in the provision of pathogen-directed treatment from .40 to .50, we required the sample size to be 470 per arm. Similarly, to detect a reduction of 0.2 SD in the time to provision of pathogen-directed treatment, we established the sample size to be 477 per arm (ie, 954 in total). Allowing for a 10% dropout rate resulted in a total sample size of 1060 patients.

Baseline patient characteristics were summarized using counts, percentages, and totals for categorical variables and medians and IQRs for continuous variables. Missing baseline values were imputed by means of a single imputation using chained equations.^15^

The 2 primary outcomes were analyzed according to the intention-to-treat principle, and Bonferroni adjustment was applied. Available-case analyses were used for the secondary outcomes.

For binary outcomes, logistic regression models with logit and identity link functions were used to estimate odds ratios (ORs) and absolute risk differences, respectively. Two models were fitted for the event-time primary outcome: Cox proportional hazards regression model and restricted mean survival time model.^16^ The proportional hazards assumption for the Cox regression model was assessed visually using cumulative log-log plots. The restricted mean survival time model is a flexible survival analysis model that does not require proportional hazards because it is based on survival curves, which may be estimated parametrically or nonparametrically.^17^ Kaplan-Meier survival curves and log-rank tests were also reported. For continuous secondary outcomes, linear regression with logarithm-transformed outcomes was used to estimate differences in medians and ratios of medians; differences in medians were approximated using a Taylor expansion.^18^ For the 2 primary outcomes, a post hoc analysis that was adjusted for season was also carried out through inclusion of an indicator of whether recruitment took place from September 25, 2020, to June 1, 2021, or from August 15, 2021, to June 21, 2022.

A significance level of *P* = .05 was applied, and the statistical software R version 4.1.2 (R Core Team) was used.^19^ Imputation through chained equations and fitting of restricted mean survival time models were carried out using the R packages mice and SURVRM2, respectively.

## Results

The trial was stopped earlier than planned because an ad hoc interim analysis that we conducted due to slow recruitment, carried out June 16, 2022, showed substantial differences between the intervention and standard-of-care arms for both primary outcomes. A total of 2265 patients were assessed for eligibility, of whom 374 participated, with 187 patients randomized to each arm (Figure 1). Both arms showed similar distributions of patient characteristics (Table 1). Patients included 153 females (40.9%) and 221 males (59.1%), with a median (IQR) age of 72 (60-79) years. Among these patients, 208 had a diagnosis of CAP, of whom 200 (97 in the intervention arm and 103 in the standard-of-care arm) provided an LRT sample. Baseline characteristics for CAP patients only (n = 200) are provided in eTable 4 in Supplement 2.

**Table 1.** Baseline Characteristics for All Randomized Patients^a^

Characteristic	No. (%)	
	Intervention arm (n = 187)	Standard-of-care arm (n = 187)
Age, mean (SD), y	73 (61-79)	71 (60-80)
Sex		
Female	73 (5.90)	80 (42.8)
Male	114 (61.0)	107 (57.2)
Current smoker	37 (19.8)	36 (19.3)
Annual influenza vaccine	97 (51.9)	98 (52.4)
Pneumococcal vaccine in past 5 y	74 (39.6)	73 (39.0)
Duration of symptoms prior to admission, median (IQR), d	6.6 (3.6-10.5)	5.7 (3.5-9.5)
Antibiotics within 48 h	42 (22.5)	37 (19.8)
Antibiotics within past mo	42 (22.5)	73 (39.0)
Comorbidities		
Hypertension	76 (40.6)	70 (37.4)
CVD	58 (31.0)	64 (34.2)
Respiratory disease	102 (54.5)	103 (55.1)
Kidney disease	23 (12.3)	16 (8.6)
Liver disease	0	2 (1.1)
Diabetes	25 (13.4)	29 (15.5)
Immunocompromised	14 (7.5)	20 (10.7)
Cancer	20 (10.7)	11 (5.9)
CCI, median (IQR)	4 (2-5)	4 (2-5)
Observations at ED presentation		
Temperature, median (IQR), °C	37.1 (36.7-37.5)	37.0 (36.8-37.5)
Pulse rate, median (IQR), bpm	94 (79-108)	93 (83-105)
Respiratory rate, median (IQR), breaths per min	22 (20-26)	24 (20-28)
O_2_ saturation, median (IQR), %	93 (90-96)	94 (89-97)
Supplementary O_2_	15 (8.0)	11 (5.9)
BP, median (IQR), mm Hg		
Systolic	131 (117-150)	135 (118-149)
Diastolic	80 (71-89)	81 (70-90)
Laboratory and radiological results		
CRP at admission, median (IQR), mg/dL^b^	8.1 (3.1-15.9)	10.8 (4.7-19.9)
WBC count at admission, median (IQR), ×10^3^/μL^b^	10.5 (8.1-15.1)	10.7 (8.1-13.8)
Chest x-ray	180 (96.3)	174 (93.0)
Chest CT	48 (25.7)	49 (26.2)
CURB-65 score, median (IQR)	1 (1-2)	1 (1-2)
qSOFA score, median (IQR)	1 (0-1)	1 (0-1)
SARS-CoV-2 positive test result	28 (15.0)	24 (12.8)

Abbreviations: BP, blood pressure; bpm, beats per minute; CCI, Charlson Comorbidity Index; CRP, C-reactive protein; CT, computed tomography; CURB-65, confusion, uremia, respiratory rate, BP, age ≥65 years; CVD, cardiovascular disease; ED, emergency department; O_2_, oxygen; qSOFA, quick Sequential (Sepsis-Related) Organ Failure Assessment score; WBC, white blood cell.

SI conversion factor: To convert CRP to milligrams per liter, multiply by 10.

^a^ Missing data (intervention: n = 10; standard of care: n = 7) were imputed using multiple imputations through chained equations.

^b^ The CRP and WBC counts were the highest values during hospitalization.

### Findings for All Randomized Patients

**Figure 2.**
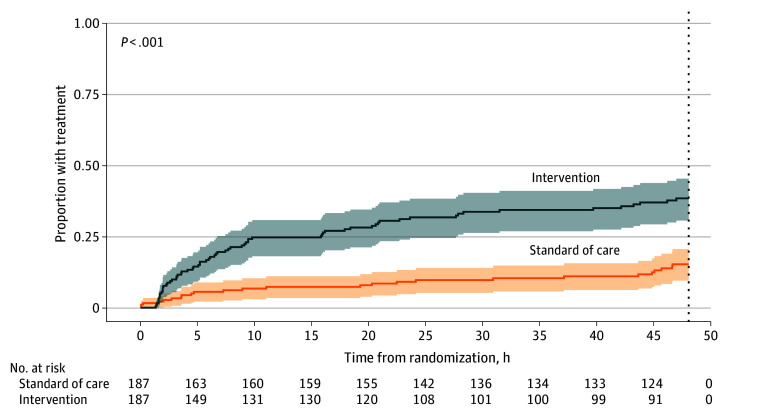
Kaplan-Meier Curve of the Proportion of Patients Receiving Pathogen-Directed Treatment The dotted line at 48 hours indicates the censoring threshold for the primary outcome of time to pathogen-directed treatment.

Forty-eight hours after randomization, 66 of 187 (35.3%) patients in the intervention arm and 25 of 187 (13.4%) patients in the standard-of-care arm received pathogen-directed treatment (Figure 2), corresponding to a reduction in absolute risk of 21.9 (95% CI, 13.5-30.3) percentage points and an OR for the intervention arm of 3.53 (95% CI, 2.13-6.02; *P* < .001) (Table 2).

**Table 2.** Comparison of Rapid Testing for All Randomized Patients by Treatment Arm

Primary outcome	Intervention arm, No. (%) (n = 187)	Standard-of-care arm, No. (%) (n = 187)	Intervention vs standard of care^a^					
			Unadjusted			Adjusted for season (2020 vs 2021)		
			Difference, % (95% CI)	Ratio (95% CI)	*P* value	Difference, % (95% CI)	Ratio (95% CI)	*P* value
Binary outcomes								
Provision of pathogen-directed treatment	66 (35.3)	25 (13.4)	21.9 (13.5 to 30.3)	OR: 3.53 (2.13 to 6.02)	<.001	22.1 (13.8 to 30.5)	OR: 3.54 (2.13 to 6.02)	<.001
Provision of any antibiotics	159 (85.0)	157 (84.0)	1.1 (−6.3 to 8.4)	OR: 1.09 (0.62 to 1.91)	.78	0.9 (−6.4 to 8.1)	OR: 1.09 (0.62 to 1.91)	.77
Event-time outcomes								
Time to provision of pathogen-directed treatment, median (IQR), h^b^	34.5 (31.6 to 37.3)	43.8 (42.0 to 45.6)	−9.4 (−12.7 to −6.0)	HR: 3.08 (1.95 to 4.89)	<.001	−9.4 (−12.7 to −6.0)	HR: 3.08 (1.95 to 4.89)	<.001

Abbreviations: HR, hazard ratio; OR, odds ratio.

^a^ Differences were estimated as absolute differences for binary outcomes (on use) and mean differences for event-time outcomes using logistic regression (with the identity link function) and restricted mean survival time methods, respectively. The ORs for binary outcomes and HRs for event-time outcomes were estimated using logistic regression and Cox regression, respectively. *P* values corresponding to testing ratios equal to 1 were reported.

^b^ Time elapsed from randomization to provision of pathogen-directed treatment within 48 hours.

The median (IQR) time to provision of pathogen-directed treatment within 48 hours was 34.5 (31.6-37.3) hours in the intervention arm and 43.8 (42.0-45.6) hours in the standard-of-care arm (mean difference, −9.4 hours; 95% CI, −12.7 to −6.0 hours; *P* < .001). The corresponding hazard ratio (HR) for the intervention group compared with the standard-of-care group was 3.08 (95% CI, 1.95-4.89). These findings remained significant after adjustment for season.

### Findings for Patients With CAP

#### Primary End Points

In the intervention arm, 46 Forty-six of 97 patients (47.4%) with CAP in the intervention arm and 16 of 103 patients (15.5%) with CAP in the standard-of-care arm received pathogen-directed treatment within 48 hours (absolute risk difference, 31.9 percentage points; 95% CI, 19.7-44.0 percentage points; *P* < .001) (Table 3), corresponding to an OR for the intervention arm of 4.90 (95% CI, 2.57-9.77; *P* < .001). The median (IQR) time to provision of pathogen-directed treatment was 29.9 (25.9-34.1) hours in the intervention arm and 42.3 (39.5-45.1) hours in the standard-of-care arm (mean difference, −12.3 hours; 95% CI, −17.3 to −7.3 hours; *P* < .001) and a corresponding HR for intervention arm compared with standard-of-care arm of 3.45 (95% CI, 1.98-6.02).

**Table 3.** Comparison of Rapid Testing for Patients With Community-Acquired Pneumonia Only by Treatment Arm

	Intervention arm, No. (%) (n = 97)	Standard-of-care arm, No. (%) (n = 103)	Intervention vs standard of care^a^		
			Difference, % (95% CI)	Ratio (95% CI)	*P* value
Outcomes on provision					
Any antibiotics	93 (95.9)	98 (95.1)	0.7 (−5.0 to 6.5)	OR: 1.19 (0.30 to 4.92)	.80
Pathogen-directed treatment	46 (47.4)	16 (15.5)	31.9 (19.7 to 44.0)	OR: 4.90 (2.57 to 9.77)	<.001
Continuation of appropriate empirical treatment	16 (16.5)	7 (6.8)	9.7 (0.9 to 18.5)	OR: 2.66 (1.07 to 7.33)	.03
Escalation from narrow-spectrum to more broad-spectrum treatment	14 (14.4)	4 (3.9)	10.5 (2.6 to 18.5)	OR: 4.04 (1.37 to 15.14)	.009
De-escalation from broad-spectrum to more narrow-spectrum treatment	10 (10.3)	5 (4.9)	5.5 (−1.9 to 12.8)	OR: 2.21 (0.74 to 7.52)	.14
Initiated pathogen-directed antimicrobial treatment, without prior empirical antibiotic treatment	6 (6.2)	0	6.2 (1.4 to 11.0)	NA	.01
Narrow-spectrum antibiotics within 48 h	81 (83.5)	87 (84.5)	−1.0 (−11.1 to 9.2)	OR: 0.93 (0.43 to 1.99)	.85
Single dose of antibiotics only	4 (4.3)	0	4.3 (0.2 to 8.4)	NA	.04
Antibiotics not used for more than 48 h^b^	14 (14.4)	22 (21.4)	−6.9 (−17.5 to 3.6)	OR: 0.62 (0.29 to 1.29)	.21
Treatment with intravenous antibiotics^b^	66 (68.0)	75 (72.8)	−4.8 (−17.4 to 7.8)	OR: 0.79 (0.43 to 1.46)	.46
Outcomes on duration					
Provision of any antibiotics during hospitalization, median (IQR), d	4.0 (2.9 to 6.0) (n = 93)	3.9 (2.1 to 6.1) (n = 98)	0.4 (−0.4 to 1.1)	Ratio of medians: 1.04 (0.87 to 1.25)	.63
Provision of intravenous antibiotics, median (IQR), d	3.3 (2.6 to 5.7) (n = 85)	3.1 (2.1 to 5.0) (n = 93)	0.3 (−0.5 to 1.0)	Ratio of medians: 1.08 (0.86 to 1.34)	.51
Provision of broad-spectrum antibiotics, median (IQR), d	3.8 (1.6 to 5.9) (n = 37)	3.9 (3.0 to 8.8) (n = 25)	−1.3 (−2.9 to 0.3)	Ratio of medians: 0.68 (0.42 to 1.10)	.11
Time to administration of antibiotics, median (IQR), h	2.1 (1.3 to 3.7) (n = 93)	2.1 (1.1 to 3.8) (n = 98)	0.26 (−0.60 to 1.12)	Ratio of medians: 1.14 (0.90 to 1.44)	.55
Turnaround time, median (IQR), h	4.0 (3.6 to 4.5)	68.2 (38.3 to 95.0)	−53.8 (−48.7 to −59.5)	Ratio of medians: 0.07 (0.06 to 0.08)	<.001
Event-time outcomes					
Time to provision of pathogen-directed treatment, median (IQR), h^c^	29.9 (25.9 to 34.1)	42.3 (39.5 to 45.1)	−12.3 (−17.3 to −7.3)	HR: 3.45 (1.98 to 6.02)	<.001

Abbreviations: HR, hazard ratio; NA, not available; OR, odds ratio.

^a^ Differences were estimated as absolute differences for binary outcomes (on use) and differences in medians for continuous outcomes (on duration) using unadjusted logistic and linear regression with logarithm-transformed outcomes, respectively. The ORs for binary outcomes and ratios of medians for continuous outcomes were estimated using unadjusted logistic and linear regression with logarithm-transformed outcomes, respectively. *P* values corresponding to testing ratios equal to 1 were reported except for 1 outcome (single dose of antibiotics) where it corresponds to testing the risk difference equal to 0.

^b^ Within the first 7 days after study inclusion.

^c^ Time elapsed from randomization to provision of pathogen-directed treatment within 48 hours.

#### Secondary End Points

The turnaround time was significantly shorter for the intervention group than for the standard-of-care group (difference, −53.8 hours; 95% CI, −48.7 to −59.5 hours; *P* < .001). Pathogen-directed treatment within 48 hours resulted in an escalation to more broad-spectrum antimicrobial treatment in 14 of 97 patients (14.4%) in the intervention arm and 4 of 103 patients (3.9%) in the standard-of-care arm (absolute difference, 10.5 percentage points; 95% CI, 2.6-18.5 percentage points; *P* = .009). Similarly, empirical antibiotic treatment was de-escalated to more narrow-spectrum treatment for 10 of 97 patients (10.3%) in the intervention arm and 5 of 103 patients (4.9%) in the standard-of-care arm (absolute difference, 5.5 percentage points; 95% CI, −1.9 to 12.8 percentage points; *P* = .14). Continuation of appropriate empirical treatment occurred in 16 of 97 patients (16.5%) in the intervention arm and 7 of 103 (6.8%) in the standard-of-care arm. This resulted in an absolute difference of 9.7 percentage points (95% CI, 0.9-18.5 percentage points; *P* = 0.03) and an OR of 2.66 (95% CI, 1.07-7.33). Additionally, 4 of 97 patients (4.3%) in the intervention arm and none in the standard-of-care arm received only a single dose of antibiotics (absolute difference, 4.3 percentage points (95% CI, 0.2-8.4 percentage points; *P* = 0.04). Further details on the comparison of rapid testing using FAP plus vs standard of care for patients with CAP are presented in Table 3. A breakdown of pathogen-directed treatment within 48 hours for patients with CAP is provided in eTable 5 in Supplement 2. For the secondary outcomes on the provision of narrow-spectrum antibiotics within 48 hours; antibiotics use for no more than 48 hours; treatment with intravenous antibiotics; and duration of provision of antibiotics, intravenous antibiotics, and broad-spectrum antibiotics, no significant differences were found.

### Length of Stay and Clinical Outcomes

Median (IQR) LOS was 3.3 (2.0-6.0) for the intervention arm and 3.2 (2.0-6.0) days for the standard-of-care arm (difference, 0.15 days; 95% CI, −0.55 to 0.85 days; *P* = .67) (eTable 6 in Supplement 2). For clinical outcomes, 29 of 187 patients (15.5%) in the intervention arm and 35 of 187 patients (18.7%) in the standard-of-care arm were readmitted (absolute risk difference, −3.2 percentage points; 95% CI, −10.8 to 4.4 percentage points; *P* = .41). Nine patients (4.8%) in the intervention arm and 7 patients (3.7%) in the standard-of-care arm died within 30 days (absolute risk difference, 1.1 percentage points; 95% CI, −3.0 to 5.2 percentage points; *P* = .61), and 16 (8.6%) and 11 (5.9%) patients in the intervention and standard-of-care arms, respectively, died within 90 days (absolute risk difference, 2.7 percentage points; 95% CI, −2.6 to 7.9 percentage points; *P* = .32).

### Microbiological Detections and Adverse Events

The intervention arm compared with standard-of-care arm had a higher number of bacterial detections (175 vs 72) and viral detections (74 vs 63). When considering only the patients with confirmed CAP, the intervention arm maintained a higher total number of bacterial (113 vs 57) and viral (39 vs 34) detections than the standard-of-care arm. The breakdown of microbiological detections in the 2 arms is shown in eTable 7 in Supplement 2.

No serious adverse events were observed, and the number of adverse events was similar in both arms for saline-induced sputum: 7 were registered in the intervention arm and 8 in the standard-of-care arm. These adverse events were dyspnea (n = 6), rapidly resolved hypoxemia (n = 5), nausea (n = 1), coughing (n = 1), and nonsevere tachycardia (n = 2). One patient in the intervention group experienced coughing during endotracheal aspiration.

## Discussion

This randomized clinical trial demonstrated that use of the FAP plus PCR test for LRT pathogens as part of the diagnostic workup for hospitalized patients with suspected CAP increases the provision of and reduces the time to pathogen-directed treatment compared with comprehensive standard-of-care microbiological testing. The FAP plus PCR test provides ED clinicians with close to real-time information for actionable treatment decisions.

To our knowledge, this trial was the first to examine the effect of a rapid syndromic PCR pneumonia panel applied specifically to patients hospitalized with CAP. Most previous studies did not use a comprehensive syndromic PCR panel or included patients shortly after admission, potentially limiting the advantages of rapid molecular testing. Another aspect of its novelty is the emphasis on pragmatism whereby decisions to continue, switch, or discontinue antimicrobial treatment were at the discretion of the treating physician alone.

The intervention led to a reduction (by 9.4 hours) in the median time without provision of pathogen-directed treatment within the first 48 hours after randomization, compared with standard of care. For patients with CAP, the median turnaround time (from admission to receiving an LRT test result without restriction to 48 hours) was reduced by much more (53.8 hours) for the intervention vs standard-of-care group. This result partly reflects hospital practice and is comparable to previous findings.^12^ A faster microbiological diagnosis allows for directed therapy, which has been shown in previous studies to improve outcomes, limit antibiotic overuse, and prevent antimicrobial resistance.^6,10^ Despite crowded conditions at the ED, a FAP plus PCR test result was delivered within 4 hours for patients with CAP, a turnaround time comparable to that achieved in other centers.^12^

No significant differences in clinical outcomes were observed between the intervention and standard-of-care groups. However, the primary objective of this trial was to enhance diagnostic stewardship by determining and then leveraging the rapid, multipathogen detection capabilities of the FAP plus PCR test.

We sought to reduce the time to provision of pathogen-directed treatment, potentially decreasing unnecessary or broad-spectrum antibiotic use and fostering antimicrobial stewardship. Future research should continue to explore innovative approaches to improving the diagnosis and management of respiratory infections, such as incorporating clinical decision support tools and antimicrobial stewardship programs into routine practice.

### Strengths and Limitations

The strengths of this study include the pragmatic design, primary outcome values obtained from electronic medical records by treating physicians who were not involved in the study, duration of 2 winter seasons, broad inclusion criteria representing typical patients with respiratory tract symptoms admitted to Norwegian hospitals, representative sampling from the LRT, a simple intervention, and comparison of intervention to standard-of-care microbiological testing. These factors suggest that the primary study findings are generalizable to similar hospital settings. The standard-of-care diagnostic testing in this study encompassed a wide battery of tests, including in-house molecular tests, rendering standard of care as competitive as possible compared with the commercial FAP plus PCR test. Moreover, the FAP plus PCR test includes several bacterial and viral pathogens along with targets for selected antimicrobial resistance genes, making this panel applicable to other settings with different microbial etiological profiles and background resistance rates for CAP.^20^

A key study strength is that the study physicians were not involved in the treatment of patients. The restriction to a narrow time frame (48 hours after randomization), which spanned the period from respiratory sampling to availability of results, supports interventions that can contribute to the timely administration of appropriate antibiotics, a central tenet of care for patients with pneumonia. The higher rate of continuation of appropriate empirical treatment for patients with CAP in the intervention arm suggests that this diagnostic tool assists in confirming the appropriateness of the initial empirical therapy and prevents unnecessary changes in treatment. Additionally, a larger proportion of patients in the intervention arm received only a single dose of antibiotics, indicating the potential for reduced antibiotic exposure.

More patients in the intervention arm than in the standard-of-care arm had an escalation from narrow-spectrum to broad-spectrum antibiotics. This escalation could raise concerns about antibiotic overuse; however, it is pertinent to emphasize that Norway has a low level of antibiotic resistance, and guidelines recommend using narrow-spectrum antibiotics.^21^ Per the guidelines, empirical treatment is benzylpenicillin for mild to moderate CAP and benzylpenicillin and gentamicin for severe CAP.^21^ Treatment with narrow-spectrum antibiotics for respiratory tract infections is common practice in Norway,^22^ leaving little room for de-escalation, and even change from penicillin G to a more broad-spectrum penicillin (eg, ampicillin) was considered to be an escalation. A background of a low level of antibiotic resistance implies that the differences found between the 2 arms for escalation or de-escalation of an antibiotic are, in a sense, the minimal differences to expect when introducing rapid testing in a Norwegian hospital setting.

This study has some limitations. First, the single-center design limits generalizability. However, the study demonstrated that embedding comprehensive rapid testing in a busy ED setting is possible. Second, the trial was stopped early for efficacy, and there could be a risk of inflated estimates of differences between the intervention and standard of care, although this risk is presumably small as we found highly significant differences. Moreover, inflation is generally small, and continuing a trial to achieve a slight change in estimates would not be rational.^23^

## Conclusions

In this randomized clinical trial, use of the FAP plus PCR test led to faster and more targeted microbial treatment for hospitalized patients with CAP. The findings align with the broader concept of clinical management or treatment stewardship for LRT infections. Routinely deployed rapid syndromic testing could complement or replace targeted components of the standard laboratory-based diagnostic repertoire for patients who are admitted to the hospital with an acute respiratory illness. Future studies should examine the effect of comprehensive rapid syndromic testing on clinical outcomes, the cost-effectiveness of this diagnostic tool, and the development of implementation strategies that facilitate the integration of rapid syndromic testing into routine clinical practice.

## Notes

Supplement 1.
Trial Protocol

Supplement 2. **eTable 1.** Targets Included in the BIOFIRE FILMARRAY Pneumonia plus Panel **eTable 2.** Standard Comments for Detected Bacteria **eTable 3.** Definitions and Diagnostic Clinical Criteria for CAP **eTable 4.** Baseline Characteristics for CAP Patients Only (n=200) **eTable 5.** Pathogen-Directed Treatment Within 48 Hours for Patients With Pneumonia (n=200) **eTable 6.** Comparison of Length of Stay and Clinical Outcomes Between Rapid Testing by FAP Plus and Standard-of-Care for All Randomized Patients (n=374) and CAP Patients only (n=200), Respectively **eTable 7.** Microbiological Detections in Respiratory Samples in the Standard-of-Care and FAP Plus Arms

Supplement 3.
Data Sharing Statement
