# Error in Figure 1

**DOI:** 10.1001/jamanetworkopen.2025.8665

**Published:** 2025-04-01

In the Original Investigation titled “Diagnostic Stewardship in Community-Acquired Pneumonia With Syndromic Molecular Testing: A Randomized Clinical Trial,”^1^ published on March 6, 2024, there was an error in exclusions shown in Figure 1. The second line should be “1080 Fulfilled <2 criteria for suspected CAP or alternative diagnosis more likely.” The article has been corrected.
