# Supplementary material for: Diagnostic Stewardship in Community-Acquired Pneumonia With Syndromic Molecular Testing: A Randomized Clinical Trial
Source: JAMA Netw Open. 2024 Mar 6;7(3):e240830. doi: 10.1001/jamanetworkopen.2024.0830 (PMC10918504; doi:10.1001/jamanetworkopen.2024.0830)
Supplement: Supplement 1. — Trial Protocol [file jamanetwopen-e240830-s001.pdf]

Protocol Title: **Impact of Molecular Testing on Improved Diagnosis, Treatment and Management of CAP in Norway: a pragmatic Randomised Controlled Trial**

Norwegian title: **Molekylær diagnostikk ved akutte luftveisinfeksjoner - konsekvenser for behandling og antibiotikaveiledning: en pragmatisk randomisert kontrollert studie**

Short Title: **CAPNOR**

Funding agencies:
Research council of Norway,
Trond Mohn Research Foundation,
University of Bergen,
Haukeland University hospital

August 2019, rev. December 2019, rev. July 2020; rev. 16092021

1.Protocol Summary
1.1 Study Rationale
1.2 Background
2. Objectives and Endpoints
3. Study Design
3.1. Number of Participants
3.2. Recruitment and triage (inclusion and exclusion criteria)
3.3 Randomisation, allocation, concealment, and blinding
3.4 Treatments/procedures
3.5 Sample size estimation
4. Assessments
4.1 Nutritional and physical status assessments at admission
4.2 Biomarker (BM) assessment of CAP aetiology and treatment outcome
5. Safety
6. Adverse events
7. Health economics
8. Statistical considerations
8.1. Primary endpoints
8.2. Secondary endpoints
8.3 Tertiary/exploratory endpoints
9. Informed consent process
10. Data protection
11. Data Quality assurance
12. Source documents
13. Study Start and Closure
14. Data management
15. Publication policy and dissemination
16. Participant Discontinuation/Withdrawal from the Study
17. Regulatory and Ethical Considerations
18. Financial disclosure
19. References
20. Appendices
1. Schedule of activities

Protocol Title: Impact of Molecular Testing on Improved Diagnosis, Treatment and Management of CAP in Norway: a pragmatic Randomized Controlled Trial

Short Title: CAPNOR

Summary

Community acquired pneumonia (CAP) causes a considerable burden of disease, which in developed countries, is greatest in the elderly. CAP is associated with excess acute and long-term mortality. As populations age, the burden of disease due to CAP will rise significantly, placing substantial strains on public health resources. Little is known about the aetiology and prevalence of CAP in Norwegian populations. Establishing clinical and etiological diagnosis is difficult and often complicated by comorbidity and the low sensitivity of routine microbiological tests. Uncertainty in diagnosis often leads to incorrect treatment and unnecessary use of broad- spectrum antibiotics. Antibiotic resistance is one of the greatest threats to human health and is driven partly by the overuse of antibiotics. Empirical antibiotics are prescribed to hospitalised patients with acute respiratory illness, including patients where viruses are strongly implicated as the cause. Interventions to reduce excessive and increase effective antibiotic prescribing may have a positive impact on antimicrobial resistance (AMR), hospital acquired infections, and clinical outcomes.

CAPNOR brings together a multidisciplinary research team from Norwegian (Haukeland University Hospital [HUS], University of Bergen [UoB], Drammen Hospital [DH], University of Oslo [UoO]) and international institutions (Denmark, Netherlands and UK), with a strong record in respiratory disease research, with the goal of contributing to improved guidelines for optimised diagnosis, treatment and management of CAP patients in Norway. CAPNOR, in line with the objectives of the funding agencies, addresses "*clinical research activities to help to ensure that patients receive high-quality and reliable diagnostics, treatment and rehabilitation throughout their disease trajectory*" and in particular, the thematic priorities "*antibiotic resistance*" and "*clinical research on diseases that pose major societal challenges in terms of prevalence, cost and/or complexity*" and "*effective, relevant studies of the elderly, and patients with multimorbidity*". CAPNOR emphasises "*translational research*" to address "*knowledge gaps*" in our understanding of CAP aetiology (in order to guide targeted treatment) and associated co- morbidities with the aim of better managing CAP and providing personalised treatment

To accomplish our goal, we will recruit CAP patients at HUS, Bergen, into a pragmatic randomised controlled trial (RCT) to assess if provision of ultra-rapid, high-quality accurate molecular diagnostics with direct feedback to the clinician can facilitate pathogen-directed usage of antibiotics, shorten antibiotic exposure and admission time and is safe. Additionally, transcriptional and immune marker profiling of patients will guide appropriate management through a targeted focus on the individual patient's physical capacity, nutritional status and co- morbidities. The pragmatic design of this trial together with broad inclusion criteria and a straightforward intervention would make our results generalisable to other similar centres.

In brief, CAPNOR brings together research groups with a strong record in respiratory and infectious disease
………..

August 2019, rev. December 2019, rev. July 2020; rev. 16.09.2021

research, with the shared goal of contributing to improved treatment, optimized management and rehabilitation of CAP patients in Norway. CAPNOR will contribute to a) impact on diagnostic thinking b) impact on therapeutic actions c) impact on patient outcomes d) impact on societal outcomes.

1.1. Rationale

Community acquired pneumonia (CAP) causes a considerable burden of disease, especially in the elderly. CAP is a serious infection and antibiotics are given at diagnosis. The treating doctor does not initially know what microbe is responsible and prescribes the patient a broad- spectrum antibiotic. The treatment is subsequently tailored to the results from the microbiology Lab., when these become available (which can take up to 48 hrs). Uncertainty in diagnosis often leads to initial incorrect treatment and unnecessary use of broad-spectrum antibiotics. An intervention that can increase targeted antibiotic prescribing could have a positive impact on antimicrobial resistance, hospital infections, and patient outcomes. We will recruit CAP patients at Haukeland University hospital, Bergen, and assess if providing ultra- rapid, accurate molecular diagnostics to CAP patients can increase the usage of targeted antibiotics that are appropriate for treating the causative microbe, shorten the exposure to antibiotics and time the patient is admitted in hospital. Further, we will attempt to guide appropriate management through a focus on the individual patients's physical capacity, nutritional status and co-morbidities.

1.2 Background

Lower respiratory tract infections (LRTI), including CAP, are the 3rd most common cause of death globally, and the second most frequent reason for years of life lost1,2. In Europe, CAP is the leading cause of death from infection, with 90% occurring in people >65 years. Pneumonia places a burden on healthcare resources, with associated annual costs in Europe estimated at > €10 billion, mainly due to hospitalisation3. Significant as this burden is, it probably represents an underestimate: CAP patients have high risk of subsequent cardiac complications and hospitalisation4, which is rarely captured in analyses of the burden of CAP disease.
Due to diagnostic difficulties, the incidence of CAP is poorly documented, even in the developed world, but the available data are consistent with an estimated figure of 535 per 100.000 persons/year, for Norway5. Mortality rates are typically 2%–14%, though both the attack rate and mortality increase with age6,7. In a recent small prospective study conducted at Drammen Hospital (DH), Norway, we found that 1-and-5-year mortality rates were 8.9% and 27.1%, respectively, among hospitalised CAP patients8 which is comparable to patients with coronary heart disease (>10%). Risk factors for readmission in patients with CAP have not been well described. Most research has focused on factors which reflect the initial severity at admission and *not* factors present prior to the pneumonia episode such as poor physical capacity, poor nutritional status, hyperglycaemia, high age and co-morbidities. By combining assessments of physical capacity, nutritional assessments and co-morbidities, and intervening where needed, we can improve management of CAP with a focus on the individual patient's risk profile.

In theory, the clinical diagnosis of CAP is based on presentation with fever, cough, sputum production, infiltration and consolidation in the lung (the latter is hampered by suboptimal sensitivity of a chest radiograph to detect pulmonary infiltrates). In practice, however, elderly patients (majority of admissions) present without many of these features9. An aetiological CAP diagnosis based on routine microbiological testing is achieved in ~ 30-40% of cases; possibly since most patients are started on empirical antibiotics prior to sampling2,8 and a significant proportion of patients initially treated for CAP do not have a lung infection9. In Norway, data on the aetiology of CAP in hospitalised patients is limited to a few small studies,

which do not include a comprehensive assessment of viral and bacterial respiratory pathogens10,11. The consequence of unsatisfactory aetiological diagnosis is that pathogen-directed therapy needs to be based on empirical standards, leading to antibiotic overuse and even abuse, especially in patients with viral infections. Implementing rapid and extensive molecular testing is likely to improve both time-to- recognised-pathogen as well as the proportion of positive tests. Both measures are important for initiation of pathogen-targeted antibiotics. Such an approach could lead to either de-escalation or escalation of treatment, where appropriate.

The lack of microbiological identification is a major problem and enhancing the detection of significant pathogens could have a major impact on the clinical decision-making process, by providing the physician with real-time information for treatment decisions. Rapid molecular testing for CAP aetiology may reduce unnecessary antibiotic use, shorten length of hospital stay, improve influenza detection and treatment, and rationalise isolation facility use and improve clinical care of CAP patients; however, limited evidence exists to support its use over standard care. A recent RCT on patient outcomes14 (conducted by a CAPNOR partner), evaluated the use of molecular testing for respiratory viruses (only) in hospitalised CAP patients in UK. Molecular testing was not associated with a reduction in the proportion of patients treated with antibiotics, probably because many patients were treated with empirical antibiotics at admission. However, molecular testing did lead to a greater proportion of patients being treated with brief courses of antibiotics, reduced length of stay and improved antiviral use and was considered safe14. These findings need replication in other studies that include rapid molecular tests targeting both viral and bacterial pathogens, and with alternate outcome measures.

In a retrospective study including positive samples from culture and rapid molecular diagnostics, Gadsby *et al.*, posited that de-escalation from broad-spectrum to pathogen-directed antibiotics could be done in 77% of patients with CAP, while escalation could be done in 6%13. Recent, large studies using molecular diagnostic tests show that respiratory viruses are detectable in ~ 40% of hospitalised adults with acute respiratory illness14. Multiplex real-time PCR can reduce the turnaround time compared with standard PCR panels from 12–30h to 2h with possible early implications on antimicrobial choice18,19. The new generation BioFire® FilmArray® Pneumonia ***plus*** platform (Biomérieux), includes the automated detection of 34 of the most relevant bacterial (including atypical bacteria) and viral respiratory pathogens, antimicrobial resistant genes (*mecA/C and MREJ, KPC, OXA48-like, NDM, CTX-M, VIM, IMP*), and semi- quantitative information on 15 common colonizers (pathobionts) to aid in presumptive differentiation between colonisation and infection. The assay requires a **20-min hands-on time and a run time of < 1hr.**

Comorbidity assessment: Malnutrition is common among patients with CAP, even in high- income countries (~20-50%)20, and the majority lose muscle mass during admission21 – with negative impact on quality of life and risk for readmission. Despite increased focus on mobilization, physical capacity (physical activity and muscle strength) is poorly monitored. Hospitalisation means bed rest with negative consequences on body composition and glucose metabolism22,23, and subsequent risk for morbidity, and mortality24. Physical capacity assessed at admission and at discharge will provide data on inactivity and loss of function. Assessment of pre- diabetes and diabetes is of interest, due to an association with mortality, longer hospital stay, admission to the ICU, and a need for transitional or nursing home care after discharge25. In CAP, newly detected hyperglycaemia increases short-term mortality26. CAPNOR will assess for pre- diabetes, known/unknown/new onset diabetes, and transient hyperglycaemia and tailor findings for individual patient

management. CAPNOR will also assess for chronic lung disease with a focus on chronic obstructive pulmonary disease (COPD), asthma, bronchiectasis and fibrosis. Patients with COPD are likely to benefit from rapid antibiotic de-escalation since they often have frequent hospitalizations and, empiric treatment usually is with a broad-spectrum antibiotic. Moreover, they often experience worsening of their condition for reasons not related to infections (e.g. heart failure) and thus, are not in need of antibiotics. Further, COPD- and cardiovascular mortality is increased the first year after an admission for CAP4a.

Exploratory immunology - diagnostic biomarkers and correlates that predict outcome in CAP An ideal biomarker (BM) should achieve rapid diagnosis, have a prognostic value and facilitate therapeutic decisions. Prognostic BMs could facilitate selection of patients who could benefit from specific therapies and thus, allow a transition from current bundled care to more personalized predict clinical outcome. These measures will be pursued in nested case-control studies, associating the early disease induced events with treatment endpoints and cross- sectional comparisons of the (immunological) phenotype and (transcriptional) genotype associated with a favourable or poor disease outcome in CAP patients. By using respiratory tract (RT) samples, whole blood, serum, and plasma (stored at the Biobank, Haukeland, Bergen) examined in nested case-control studies, we will apply the best knowledge at the time of study conclusion, thus assessing the most relevant markers with the most relevant assays. Based on the present state of knowledge, for transcriptional profiling we will use a dual-color Reverse- Transcriptase Multiplex Ligation-dependent Probe-Amplification (dcRT-MLPA) assay and/ or by RNA sequencing (RNAseq) 27-29 using a panel of genes that target T and B cell markers as well as, type 1 interferon-inducible genes. Detection of selected recently identified classifier genes that discriminate between viral and bacterial LRTI (e.g., *IFI27, RSAD2, OAS2, IFIT3*) will be evaluated.

2. Objectives

Overall objective Among patients hospitalized with CAP:

Assess the utility of a comprehensive ultra-rapid molecular diagnostic approach compared to current standard of care on a broad range of clinical outcomes including antibiotic use

Assess patient risk profile (e.g. physical capacity and hyperglycaemia) as effect modifiers and predictors of disease outcome

Identify and evaluate novel diagnostic and prognostic biomarkers

The project objectives will be realised by first conducting a limited pilot prospective cohort study (approx. 100 patients with suspected CAP) followed by an RCT on the impact of an ultra-rapid molecular diagnostic approach compared to current standard of care on a broad range of clinical outcomes.

The pilot study will mirror the procedures described below for the RCT but, will not include the randomization of respiratory samples to two treatment arms (as described for the RCT). Thus, for the pilot

study all respiratory samples will be tested by standard of care microbiological testing and by comprehensive ultra-rapid molecular testing (UR-MT). The limited pilot study will be used to assess several parameters, including the feasibility of recruitment, an assessment of the included clinical and laboratory procedures, and the accuracy and efficiency of the data entry process, data transfer and data analysis using the study-specific eCRF. Finally, the pilot study would inform on any procedural or design refinements needed for the ensuing CAP RCT.

**Primary objective**
In hospitalised community-acquired pneumonia (CAP) patients, conduct an RCT on the impact of an ultra-rapid molecular test (UR-MT) versus standard of care on the i) time to receiving microbiology testing-informed treatment and ii) the provision of pathogen-directed treatment based on a microbiological test result.
**Primary outcomes:** The impact of the UR-MT will be assessed by two outcomes: i) time to receiving microbiology testing-informed treatment and 2) the provision of pathogen-directed treatment based on a microbiological test result. Establishing an effect of the ultra-rapid test on either outcome will be considered to be clinically important.
**Secondary objectives**
• Determine the etiology of community-acquired pneumonia (CAP) in hospitalised patients<br>• Determine the association of positive tests with severe and very severe pneumonia and determine the attributable fraction of pneumonia cases associated with a defined aetiology or mixed infections<br>• Assess the prevalence of nutritional deficiencies, hyperglycaemia, physical capacity and their role as effect modifiers and predictors of outcome in CAP patients<br>• Assess the association between nutritional status, physical and functional capacity during admission on length-of-stay, length-of-antibiotic-treatment, risk of re-admission and mortality<br>• Identify and evaluate biomarkers of CAP aetiology<br>• Identify and evaluate correlates of disease evolution and treatment outcome

Secondary outcomes linked to the primary objective: To capture the entire range of clinically relevant antibiotic and non-antibiotic changes associated with the UR-MT, we will assess: duration of antibiotic use; proportion of patients receiving a single dose of antibiotics; proportion of patients receiving ≤48 h of antibiotics; proportion of patients receiving intravenous (IV) antibiotics; duration of IV antibiotics; time to specimen receipt; proportion of cases where the UR-MT results were not used to guide treatment; proportion of patients with influenza treated with NI; time to NI use; duration of NI use; time to isolation or de-isolation; "door-to-needle time"; length of hospital stay (LOS), 30-day readmission, 30- and 90-day mortality, and 1- and 5-year mortality (data will be obtained from the Norwegian Cause of Death Registry).

3. Overall Design

The study is a pragmatic, single-blind, single-centre randomised controlled trial (RCT) where CAP patients will receive standard of care microbiological testing or standard of care testing and comprehensive ultra-rapid molecular testing (UR-MT).

3.1 *Number of Participants*

We will over a 3-year period (2019-2022), consecutively enrol cases of CAP admitted (~900/year) to Haukeland University Hospital (HUS, Bergen). HUS is a large teaching hospital that serves as a local hospital for a population of ~ 350.000. At admission to the emergency department (ED) i.e. the recently (2014) organised "Mottaksklinikken" at HUS. The study will consist of representative patients admitted with CAP and thus, is potentially generalisable to hospitalised patients with CAP in Norway. As, COVID-19 cannot be distinguished clinically from other pneumonias, the study will therefore include patients with suspected CAP, including with COVID-19. Approximately 2500 CAP patients will be screened to achieve a total of 1390 (allowing for a 10% dropout rate) enrolled patients that are randomly assigned to receive standard of care microbiological testing or standard of care testing and the comprehensive ultra-rapid molecular test (UR-MT).

Note: "Enrolled" means a participant's, or their legally acceptable representative's, agreement to participate in the study following completion of the informed consent process (see section on informed consent as well as, the attachment on consent).

3.2 Recruitment and triage **Inclusion criteria** for the study are: adults (aged ≥18 years), with a clinical diagnosis of CAP (presence of at least two clinical criteria [new/worsening cough, new/worsening expectoration of sputum, haemoptysis, new/worsening dyspnoea, pleuritic chest pain, fever, or abnormalities on chest auscultation or percussion] or one clinical criterion and radiological evidence of CAP), requiring hospitalisation to a non-ICU ward, and with a capacity to give informed written consent or consent provided by the patient's legally authorized representative.

Potential participants who are screened for the purpose of determining eligibility for the study, but do not participate in the study, will not be considered enrolled.

………..

Exclusion criteria include: pulmonary embolism, lung tumour, cystic fibrosis, a palliative approach, patients who decline to provide respiratory tract specimens, severe immunodeficiency, and hospitalisation for two or more days in the last 14 days. Based on clinical evaluation and data on admission, patients will be triaged for severity according to current risk assessment guidelines, as well as the CRB-65 score[31] for the assessment of severity of pneumonia (see Appendix 1). Data will be entered into standardised patient-specific electronic case record forms (eCRFs).

3.3 *Randomisation, allocation, concealment, and blinding* Randomization of CAP patients to the two treatment arms (1:1) will be performed in blocks of size 4, 6, or, 8, occurring in random order, to ensure approximately equal allocation over the year. A staff member not involved in data collection will produce a computer-generated randomization list of consecutive numbers, such that each number is linked to one of the two treatments. **Treatment allocation** will take place at Dept. of Microbiology at receipt of the respiratory tract (RT) specimen. The randomization list will be generated from a prespecified seed using the extension package "blockrand" for R version
3.4.4. The order in which participants finish the baseline visit will determine their number in the randomization list (and thereby which envelope and allocation they receive). By this procedure, participants and investigators will be blinded to the allocation until the baseline measurement has been completed. However, owing to the nature of treatments, research staff, and clinical care providers will not be blinded to group allocation. Data analysts will be blinded to treatment allocation.

3.4 *Treatments/procedures* Following consent, all suspected CAP patients will be tested for their SARS-CoV-2 status based on testing of a nasopharyngeal/oropharyngeal swab sample. A confirmed case of COVID-19 will be defined as a positive result on the GeneXpert system (Xpert Xpress SARS-CoV-2) or in an in-house real-time reverse-transcriptase–polymerase-chain-reaction (RT-PCR) assay on nasopharyngeal/oropharyngeal (NP/OP) swab samples. Respiratory tract (RT) samples (see table on Schedule of activities) will be collected at admission on all patients suspected of CAP, will be sent immediately via a pipeline system to the Dept. of Microbiology, HUS. Current, hospital control recommendations will be followed for obtaining respiratory samples. In addition, blood for culture and for investigations listed in the table on Schedule of activities, as well as, urine for antigen testing (*S. pneumoniae* and *L. pneumophila*) will be collected.

Ultra-rapid molecular testing (UR-MT) comprises automated detection using the new BioFire® FilmArray® Pneumonia ***plus*** platform (Biomérieux). The **total turn-around time is <2 hrs.**

Microbiological processing per current standard of care entails culture of RT samples according to national protocols to detect respiratory bacteria, identified using biochemical methods and/or matrix-assisted laser desorption/ionization time-of-flight (MALDI-TOF MS). Respiratory viruses are identified using an in-house real-time PCR[32-37] (for metapneumovirus, rhinovirus, influenza A, influenza B, parainfluenza 1-3, RSV and SARS-CoV-2). The **total turn-around time is up to 48 hrs.**

……..

Positive feedback loop Among patients randomised to UR-MT, results (negative and positive) will be telephonically communicated to the ward the patient is admitted to at HUS, with a non-binding advice to change treatment from empirical antimicrobials to pathogen-directed therapy. Baseline institutional antimicrobial stewardship interventions will be in place for all patients.

The prescribed empirical therapy for each patient will be compared with what antimicrobial(s) would have been appropriate for pathogen-directed therapy, based on the UR-MT result. Appropriate pathogen-directed therapy will be determined using national guidelines recommended by the Norwegian directorate of health [38]. Empirical treatment as well as, pathogen specific treatment guidelines followed at Mottaksklinikken, HUS for CAP are provided at: https://helsedirektoratet.no/retningslinjer/antibiotika-i-sykehus/seksjon. For example, for empirical treatment of CAP with a CURB65 of 3-4: benzylpenicillin iv 3 g x 4 + gentamicin iv 5 mg/kg x 1* or cefotaxime 1-2 g x 3 i 7-10 days* (* in addition, a macrolide antibiotic if Mycoplasma or Legionella is suspected: Erythromycin iv 500 mg x 4 or Azithromycin iv 1 g x 1 for Legionella). Implementing rapid and extensive molecular testing is likely to improve time-to- recognised-pathogen and de-escalation or escalation of treatment, where appropriate.

Further, the decision making behind antibiotic prescribing will be explored (e.g. focus group discussions) together with the participating clinicians and clinical departments. During the ongoing phase, antibiotic prescribing will be recorded, and decisions and prescribing not in alignment with the molecular test results and/or national p rescribing recommendations, recorded and explored. The study will enable the identification of factors that hinder or promote the use of molecular diagnostics in antibiotic prescribing decisions.

3.5 *Sample size estimation*

To ensure a sufficiently large sample size to address the multiplicity issue introduced by considering two primary outcomes, we take a conservative approach of assuming that the two outcomes are uncorrelated, implying that separate sample size calculations for the two outcomes have to be at a significance level of 0.025 instead of 0.05.

In a small prospective study on hospitalised CAP patients, with stringent inclusion criteria we show that microbial aetiology with a combination of molecular and conventional methods, could be established in 62% [25]. Therefore, for the proportion of patients with change in treatment from empirical antimicrobials to pathogen-guided treatment, we expect to identify a pathogen in at least 50% of the cases using the ultra-rapid molecular test (UR-MT) versus 40% with the current standard of care. Assuming a significance level of 0.025 and a power of 0.9 the required sample will be 1226. Allowing for a 10% dropout rate results in a total sample size of 1363 assuming a power of 0.9.

For the time to change from empirical treatment to pathogen-guided treatment, we have no data from previous studies on effect size to gauge the sample size calculation and therefore define the effect size in term of the variation in the outcome (the standard deviation)[26]. We find it clinically relevant to be able to detect a difference of 0.2 standard deviation, e.g., if the standard deviation of the time to change would be 3 days then we can detect a difference of 0.2 * 3 = 0.6 day. The required sample size is 1244, assuming a significance level of 0.025 and a power of 0.9. Allowing for a 10% dropout rate results in a total sample size of 1382 patients assuming a power of 0.9.

………..

In conclusion, a sample size of 1390 will ensure that we have 90% power to detect a difference in at least one of the two primary outcomes (at a significance level of 0.025). We expect to recruit ~450-500 CAP patients per year, and therefore realistically expect to complete enrolment within 3 years.

4. Patient Assessments

4.1 ***Nutritional and physical status assessments at admission***

•Anthropometry (height, weight, skinfold thickness), changes in body composition (fat and fat- free mass) and fluid balance will be determined using the bio-impedance analyser (Maltron touch i8).

•Micronutrients important to immune function and CAP: vitamin D (25(OH)D), vitamin A (retinol binding protein) and zinc. Vitamin D regulates the production of antimicrobial peptides (cathelicidin and beta-defensin-2), which play an important role in the innate immune response to infection[41].

Data on habitual alcohol consumption and smoking will also be collected.

Physical capacity monitoring Physical capacity (muscle strength) will be measured with the handgrip test. Physical activity will be measured using three axial accelerometer-based physical activity monitors (Axivity AX3, UK). All patients will be equipped with one accelerometer at inclusion and for 7 days or until discharge. Physical capacity will be correlated to markers of disease such as blood glucose and inflammatory markers.

Diabetes status will be assessed using HbA1c[42]. All participants will be tested at admission to identify u n k n o w n /new o n s e t d i a b e t e s and pre-diabetes, to differentiate hyperglycaemia between patients with-and without diabetes (exhibit different risk profiles), and glycaemic control in known diabetes. We will assess acute dysregulation by comparing admission plasma glucose to estimated mean plasma glucose derived from HbA1c, which seems superior to admission plasma glucose alone to predict a poor outcome (preliminary results: Faurholt-Jepsen *et al*; CAPNOR partner). Admission plasma-glucose levels appear to have little value as a diagnostic marker alone but will be used for risk stratification in combination with inflammatory markers. The cut-off values for both fasting and postprandial hyperglycaemia among the patients without diabetes will be defined by receiver operator curve (ROC) analysis. In addition, we will assess impact of other pre-admission co-morbidities, such as, COPD, heart disease and kidney disease.

4.2 *Biomarker (BM) assessment of CAP aetiology and treatment outcome* Risk stratification of patients at admission is required to guide management and treatment decisions. The most established score systems (e.g. CURB-65) may accurately predict mortality in some CAP patients, but do not automatically identify patients that would benefit from aggressive management strategies. There is some evidence that BMs can improve risk stratification and management decisions in CAP: assessing procalcitonin (PCT) levels may help reduce the duration and frequency of antibiotic therapy[43] and levels of novel BMs e.g., proadrenomedullin (MR-proADM) and copeptin have been associated with 28-day and long-term mortality[44].

Recently, we have shown IL-6, IL-8 and MIP-1(beta) levels are associated with disease severity

………………………………………………………………………………………………………..

August 2019, rev. December 2019, rev. July 2020; rev. 16.09.2021

(determined using CURB-65 score), whilst IL-6 and MIP-1(beta)[45] and circulating DNA (cfDNA)[46] are associated with poor short-term outcome. Additionally, transcriptional profiling has shown superiority over assessment of PCT alone for distinguishing bacterial from viral LRTI in hospitalised adults[30].

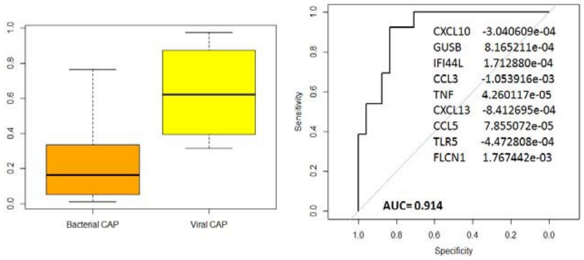

To this end, RNA samples collected in a CAP study conducted at Drammen hospital, Norway[8], were used as the discovery cohort for identification of potential diagnostic transcriptional biosignatures.

Fig 2 *Comparison of dcRT-MLPA gene expression data between "Bacterial CAP" and "Viral CAP" by LASSO regression analysis. The predicted probability of the 9-transcript signature to discriminate between Bacterial CAP and Viral CAP is shown by: box-and-whisker plots (5-95 percentiles) and receiver operating characteristic and area under the curve (ROC/AUC).*

Preliminary results (Grewal HMS *et al*; CAPNOR partner) indicate that a 9-gene transcript identified by dcRT-MLPA, could discriminate between viral and bacterial CAP, providing an AUC of 0.914 (95%CI, 0.826–1.00) [Fig. 2]. The biosignature will be evaluated in samples collected in our CAPNOR cohort (validation cohort).

Methods ***dcRT-MLPA*** the viability of using dcRT-MLPA for BM studies in clinical trial settings[48] has been demonstrated using gene panels relevant for assessment of LRTI, covering effector T cell markers, genes associated with innate and adaptive immune profiling, and IFN-inducible genes[27-29,49]. In brief, total RNA is extracted from PAXgene blood collection tubes and relative expression is determined with dcRT-MLPA assay[27], which is established at UoB, Norway[28]. ***Luminex assay (Bio-plex)*** quantification of multiple protein BMs in selected clinical samples will be assessed by the Multiplex Bead Array-Bio-Plex assay using custom designed human chemokine/cytokine kits (Bio-Rad) and measured by the Bio-Plex 200 System with Luminex xMAP technology. The utility of this technique has been validated in several of our studies[28,29,50] and complements dcRT-MLPA by allowing assessment from cell-free samples and providing confirmation that selected transcribed genes are expressed at the protein level**.** ***Immunosorbent assay (ELISA)and immunoluminometric assays*** PCT measured by a chemiluminescent assay (Brahms, Germany); MR-prōADM by an immunofluorescent assay (Brahms, Germany); copeptin measurements with an immunoluminometric assay[51]; and cathelicidin and beta-defensin-2 by ELISAs[52].

5. ***Safety***

The FilmArray® Pneumonia platform has demonstrated excellent sensitivity and specificity in recent ……..

multicentre evaluations and we consider the risks associated with this test to be low. Respiratory samples collected on admission, as part of standard of care, cause no additional risk. The risk of blood sampling in the form of pain, bleeding and infection is minimal. Treatment recommendations to escalate, de-escalate or stop antibiotics treatment may be beneficial for the individual patient by minimising exposure to antibiotics and/or improving pathogen-specific targeted use of antibiotics. Final decisions will always be made by the treating physician taking into account all clinical and diagnostic information.

6. ***Adverse events*** **(AE)**

Adverse events will be reported as per regulatory requirement. Safety outcomes will be admission to the intensive care unit while hospitalised, re-admission to hospital, and death within 30 days of enrolment. AEs will be reported by the participant (or, when appropriate, by the participant's legally authorized representative). The investigators and qualified designees will be responsible for detecting, documenting, and recording events that meet the definition of an AE and will remain responsible for following up AEs that are considered related to the study. An independent data safety monitoring board (DSMB) of health care professionals outside the study team will be established. DSMB will monitor all cases of serious adverse event. DSMB will have access to the randomization code and will not be blinded to the treatment of the subjects. The board will review safety data on a bi- annual basis and advise on relevant actions to be taken, e.g., go/no go of the trial.

7. Health Economics

Health economics data, associated with medical encounters, will be collected in the eCRF by the investigators and study-site personnel for all participants. Economic data will be computed by combining information on molecular test performances, linked to consequences for costs for diagnosis and treatment. The Dept. of Microbiology, HUS, has developed a well evaluated model for estimating the total cost of performing tests that includes reagents, technician time, instrument-cost etc. This model will be used to calculate the actual cost of performing the UR- MT (FilmArray *plus*).

The data collected will be used to conduct exploratory economic analyses and will include:

- Number and duration of medical care encounters
- Duration of hospitalization (total days or length of stay)
- Number and type of diagnostic and therapeutic tests and procedure
- Potential cost reductions related to reduced hospital stay, reduced use of isolation rooms and possibly fewer days admitted to the ICU. Other factors like reduction in use of antibiotics, and more rapid transfer to per oral treatment will also be included.

8. Statistical Considerations

Statistical Analyses

……..

August 2019, rev. December 2019, rev. July 2020; rev. 16.09.2021

The statistical analysis plan will be finalized prior to un-blinding and it will include a more technical and detailed description of the statistical analyses described in this section. This section is a summary of the planned statistical analyses of the most important endpoints including primary and key secondary endpoints.

8.1 Primary endpoints Proportion of patients with change in treatment from empirical antimicrobials to pathogen-guided treatment will be analysed by means of logistic regression. Time to change from empirical treatment to pathogen guided treatment will be analysed by means of linear models.

8.2 Secondary endpoints Secondary outcomes will be analysed using the same types of models. As it is a pragmatic trial the main statistical analyses will be carried out according to the intention-to-treat principle using appropriate effectiveness/de facto estimands for handling dropouts and missing values[40]. Additionally, efficacy may be evaluated using per protocol analyses. Statistical analyses will be done with Prism version 6.0 (GraphPad Software; La Jolla, CA, USA), R version 3, 4.4 or a later version (R Core Team, Vienna, Austria), and Stata version 13.1 (StataCorp; College Station, TX, USA).

8.3 Tertiary/exploratory endpoints Prevalence of known/new onset pre- diabetes and diabetes, nutritional deficiency, and level of physical activity/capacity during admission will be reported. Acute glucose dysregulation will be determined from linear regression by comparing admission p-glucose to estimated mean p-glucose derived from HbA1c. When evaluating outcomes in the RCT, we will test for confounding and effect modification by diabetes status, nutritional deficiencies, cognitive status, and physical capacity using the same models as described for computing primary endpoints, except that additional interaction terms are included. We will further control for confounders such as age, gender, and BMI using multiple linear and logistic regression models. Identification of biomarkers of CAP aetiology will be based on LASSO and related statistical learning methods for developing prediction models through the use of training and test datasets. Optimal tuning parameters will be determined using cross validation. Similar methods will be used to identify biomarkers that are predictive of treatment outcome. Results will be summarized using ROC curves, in terms of AUC, sensitivity, and specificity.

9. ***Informed Consent Process***

- The investigator will explain the nature of the study to the participant or his/her legally authorized representative and answer all questions regarding the study.
- Participants will be informed that their participation is voluntary. Participants or their legally authorized representative will be required to sign a statement of informed consent that meets the requirements of the IRB/IEC.
- The medical record will include a statement that written informed consent was obtained before the participant was enrolled in the study and the date the written consent was obtained. The authorized person obtaining the informed consent will also sign the informed

consent form (ICF).

- A copy of the ICF will be provided to the participant or the participant's legally authorized representative.
- The ICF will contain a separate section that addresses the use of remaining mandatory samples for exploratory research.
- Participants will be told that they are free to refuse to participate and may withdraw their consent at any time and for any reason during the storage period.

If a patient is unable to provide consent for e.g., is confused and disoriented at admission, we will obtain a) consent from the patient as soon as the acute phase of infection subsides; b) if the patient still remains unable to provide consent once the acute infection subsides, we will obtain telephonic consent from the nearest relative listed on the patient admission form. The date and time of the telephonic consent obtained, as well as the name and address of the person (nærmest pårørende) contacted will be registered in the electronic case verification form. If the patients refuses consent and/or the nearest relative cannot be contacted or refuses consent on the patients behalf, all information registered in the patient eCRF as well as, respiratory and blood samples that have been collected as per REK approved protocol will be destroyed.

10. *Data Protection*

- Participants will be assigned a unique identifier. Any participant records or datasets that are transferred will contain the identifier only; participant names or any information which would make the participant identifiable will not be transferred.
- The participant will be required to give consent for their data to be used as described in the informed consent.

11. *Data Quality Assurance*

- All participant data relating to the study will be recorded in the electronic CRF.
- The investigators are responsible for verifying that data entries are accurate and correct by physically or electronically signing the CRF.
- The investigators will maintain accurate documentation (source data) that supports the information entered in the CRF.
- The investigators are responsible for the data management of this study including quality checking of the data.
- Study monitors will perform ongoing source data verification to confirm that data entered into the CRF by authorized personnel are accurate, complete, and verifiable from source documents; that the safety and rights of participants are being protected; and that the study is being conducted in accordance with the currently approved protocol and any other study agreements, ICH GCP, and all applicable regulatory requirements.
- Records and documents, including signed ICFs, pertaining to the conduct of this study will

be retained by the investigators for 10 years after study completion.

12. *Source Documents*

- Source documents will provide evidence for the existence of the participant and substantiate the integrity of the data collected. Source documents will be filed at the investigator's site.
- Data reported on the CRF or entered in the eCRF that are transcribed from source documents will be checked for consistency with the source documents and any discrepancies will be explained.

13. *Study Start and Closure*

The study start date as stated herein, is the date on which the clinical study will be open for recruitment of participants for the proposed RCT. This is now, due to the delay caused by the COVID-19 epidemic, to start in September 2020. For this study, a sample size of 1390 will ensure that we have 90% power to detect a difference in at least one of the two stated primary outcomes (at a significance level of 0.025). We expect to recruit ~450-500 CAP patients per year, and therefore realistically expect to complete enrolment within 3 years i.e. in the last quarter of 2023. The study will close in 30.06.2028.

The investigators may initiate study-site closure at any time, provided there is reasonable cause and sufficient notice is given in advance of the intended termination. Reasons for the early closure may include but are not limited to, inadequate recruitment of participants by the investigators. If the study is prematurely terminated the investigators will promptly inform the IECs/IRBs and the contract research organizations of the reason for termination.

14. Data management

Responsibility for the data management and the contact person for questions regarding the research data are: Project PI: Professor Harleen Grewal, Dept. of Clinical Science, Univ. of Bergen and Project co-PI Professor Elling Ulvestad, Dept. of Microbiology, Haukeland Univ. Hospital. **Format:** research data will be stored in file formats that can be read with standard software tools allowing storage, reuse and accessibility to relevant users. As, Excel and Stata formats change over time, we plan to store data in a basic text format, without any or significant formatting. Such a format can always be imported into various (statistical) software programmes. Depending on the number of data files (that may have to be combined) it may be more or less demanding. **Metadata**: Research data generated by NORCAP will be accompanied by standardised metadata, that will follow international standards. The metadata will be registered in a meta-form as an xml-file. As one of several options, data files may be accompanied by lists of corresponding variable labels for convenient identification of relevant variables. **Organising data:** depending on how many sources of data we envisage; we will assign unique identifiers in all data files. We have offset expenses for data management under the CAPNOR budget. **Data storage and back-up:** the IT department at the University of Bergen has developed a solution for the handling of sensitive personal data in research. The solution is called SAFE (Secure Access to Research Data and E-infrastructure). SAFE is based on the secure handling of health and care

information and ensures that information with respect to confidentiality, integrity and availability is secured and protected during the treatment of sensitive personal information. The system is presently free of charge.

Haukeland University Hospital ensures the integrity and confidentiality of all enrolled patients by providing unique ID (identity) numbers for all person-identifiable data. All patient samples are assigned a unique sample ID (generated by UNILABS Systems). Samples from patients enrolled in the CAPNOR study will be further assigned a CAPNOR study ID. The key linking the UNILAB ID with the CAPNOR study ID will be stored on separate server that is maintained by Helse Vest IKT. At HUS, only authorised physicians employed at the Dept. of Microbiology or at the Dept. of Infectious Diseases have access to the UNILAB ID and to patient-identifiable data.

15. ***Publication Policy and dissemination***

The results of this study will be published or presented at scientific meetings. The investigators will comply with the requirements for publication of study results. Authorship will be determined by mutual agreement and in line with International Committee of Medical Journal Editors authorship requirements. The final publication plan will be approved by all partners. However, the basic assumption is of freedom to publish, and can be summarized as follows: the coordinator will compile reports after six-monthly meetings and prepare work plans based on input and discussion with the work package leader teams. These reports will be distributed together with assessments, recommendations and analysis of progress, as well as with assessments of obstacles and new opportunities.

In addition, technical bulletins on methodological updates will be disseminated to appropriate partners. These publications will be restricted to the consortium unless agreed otherwise by all involved partners (for example, to make press releases). Importantly, we will publish the results of the proposed study in peer-reviewed international journals and data will be made available in appropriate databases. The partners in this project are academic medical institutions/university hospitals, and the previous commitment of the collaborating scientists to publication is well documented. The only deviation from a policy of unobstructed publication is the requirement that before submitting results for publication, the corresponding author will circulate a draft to the partners no less than 15 days before submission. Partners who identify a legitimate commercial interest may request a delay of no more than 60 days to allow for filing of patents, in which case the rules for protection of IPR, which will be laid out in the consortium agreement will be followed. Further, users from relevant national and local organisations and societies have been included and will be involved in planning and dissemination at regular intervals e.g. relevant clinical departments at HUS, LHL (patient organisation for persons suffering from heart and lung diseases), the Norwegian Diabetes Association.

*16. **Participant Discontinuation/Withdrawal from the Study***

- A participant will be able to withdraw from the study at any time at his/her own request.
- If the participant withdraws consent, the investigators may retain and continue to use any data collected before such a withdrawal of consent.
- If a participant withdraws from the study, he/she may request destruction of any samples taken and not tested, and the investigator will document this in the study records.

………..

August 2019, rev. December 2019, rev. July 2020; rev. 16.09.2021

17. Regulatory and Ethical Considerations

This study will be conducted in accordance with the protocol and with the following:

- Consensus ethical principles derived from international guidelines including the Declaration of Helsinki and Council for International Organizations of Medical Sciences (CIOMS) International Ethical Guidelines
- Applicable ICH Good Clinical Practice (GCP) Guidelines
- Applicable laws and regulations
- Any amendments to the protocol will require IRB approval

The investigators will be responsible for providing oversight of the conduct of the study at the site and adherence to requirements of 21 CFR, ICH guidelines, the IRB/IEC, and all other applicable local regulations

18.***Financial Disclosure***

Investigators are responsible for providing information on financial interests during the course of the study and for one year after completion of the study.

19. *References* *CAPNOR partners

1. World Health Organization. The top 10 causes of death. http://www.who.int/en/news-room/fact-sheets/detail/the-top-10-causes-of-death (last accessed 24.04.2018). **2**. Jain, S. *et al. N Engl J Med* 373, 415-427, (2015). **3**. Torres, A., Peetermans, W. E., Viegi, G. & Blasi, F. *Thorax* 68, 1057-1065, (2013). **4**. Corrales-Medina, V. F. *et al. PLoS Med* 8, e1001048, (2011). **5**. Gray, S. *et al. European Respiratory Journal* 42, (2013). **6.** Welte, T., Torres, A. & Nathwani, D. *Thorax* 67, 71-79, (2012). **7.** Leoni, D. & Rello, J. *Curr Opin Infect Dis* 30, 240-247, (2017). **8*.** Holter, J. C. *et al. PLoS One* 11, e0148741, (2016). **9.** Musher, D. M. & Thorner, A. R. *N Engl J Med* 371, 1619-1628, (2014). **10*.** Holter, J. C. *et al. BMC Infect Dis* 15, 64, (2015). **11.** Roysted, W. *et al. Clin Respir J* 10, 756-764, (2016). **12*.** Brendish, N. J. *et al. Lancet Respir Med* 5, 401-411, (2017). **13.** Gadsby, N. J. *et al. Clin Infect Dis* 62, 817-823, (2016). **14*.** Clark, T. W. *et al. J Infect* 69, 507-515, (2014). **15.** McCullers, J. A. *Nat Rev Microbiol* 12, 252-262, (2014). **16.** Marzoratti, L. *et* al. Curr *Infect Dis Rep* 14, 275-283, (2012). **17.** Muthuri, S. G. *et al. Influenza Other Respir Viruses* 10, 192-204, (2016). **18.** Gelfer, G., *et al. Diagn Microbiol Infect Dis* 83, 400-406, (2015). **19.** Messakar, K. *et al* J Clin Microbiol. 55(3):715-723 (2017). **20.** Rasmussen, H. H., *et al. Clin Epidemiol* 2, 209-216, (2010). **21.** McWhirter, J. P. & Pennington, *BMJ* 308, 945-948 (1994). **22.** Bienso, R. S. *et al. Diabetes* 61, 1090-1099, (2012). **23*.** Olsen, R. H., *et al. JAMA* 299, 1261-1263, (2008). **24.** Partridge, J. S. *et al. Age Ageing* 41, 142-147, (2012). **25.** Corsino, L., Dhatariya, K. & Umpierrez, G. *Endotext* (eds L. J. De Groot *et al.*) (2000). **26.** Rueda, A. M. *et al. J Infect* 60, 99-105, (2010). **27.** Joosten, S. A. *et al. Genes Immun* 13, 71-82, (2012). **28*.** Dhanasekaran, S. *et al.Genes Immun* 14, 356-364, (2013). **29*.** Dhanasekaran, S. *et al. PLoS Negl Trop Dis* 8, e3243, (2014). **30.** Suarez, N. M. *et al. J Infect Dis* 212, 213-222, (2015). **31.** Lim, W. S. *et al. Thorax* 58, 377-382 (2003). **32.** Khanna, M. *et al. J Clin Microbiol* 43, 565-571, (2005). **33.** McDonough, E. A. *et al. Mol Cell Probes* 19, 314-322, (2005). **34.** Jokela, P. *et al. Clin Microbiol Infect* 16, 1568-1573, (2010). **35.** Tiveljung-Lindell, A. *et al. J Med Virol* 81, 167-175, (2009). **36.** Schulze, M. *et al. PLoS One* 5, e9966, (2010).
37. Lu, X. *et al. J Clin Microbiol* 46, 533-539, (2008). **38.** https://helsedirektoratet.no/retningslinjer/antibiotika-i-sykehus. **39.** Olsen, M. F. *et al. BMJ* 348, g3187, (2014). **40.** Leuchs, A. K., Brandt, A., Zinserling, J. & Benda, N. *Pharm Stat* 16, 12-19, (2017). **41.** Gombart, A. F. *Future Microbiol* 4, 1151-1165, (2009). **42.** Authors/Task Force Members, Ryden, L. *et al.* ESC *Eur*

……..

August 2019, rev. December 2019, rev. July 2020; rev. 16.09.2021

Heart J 34, 3035-3087, (2013). **43.** de Jong, E. *et al. Lancet Infect Dis* 16, 819-827, (2016). **44.** Kolditz, M. *et al. Respir Med* 106, 1320-1328, (2012). **45*.** Siljan, W. W. *et al. Eur J Clin Invest* 48, (2018). **46*.** Siljan, W. W. *et al. J Infect* 73, 383-386, (2016). **47.** Everitt, A. R. *et al. Nature* 484, 519-523, (2012). **48*.** Jenum, S. *et al. Sci Rep* 6, 38841, (2016). **49.** Berry, M. P. *et al. Nature* 466, 973-977, (2010). **50*.** Dhanasekaran, S. *et al. Genes Immun* 15, 265-274, (2014). **51.** Morgenthaler, N. G., Struck, J., Alonso, C. & Bergmann, A. *Clin Chem* 52, 112-119, (2006). **52.** Cakir, E. *et al. Int J Tuberc Lung Dis* 18, 671-675, (2014). **53*.** Jenum, S. *et al. Sci Rep* 6, 18520, (2016). **54*.** Faurholt-Jepsen, D. *et al. PLoS One* 6, e24215, (2011). **55.** Maggi, S. *Expert Rev Vaccines* 9, 3-6, (2010). **56.** Ministry of Health Labour and Welfare, Pamphlet; Available at: http://www.mhlw.go.jp/english/org/pamphlet/dl/pamphlet-about_mhlw.pdf, (2016). **57**. Postma, D.F. *et al*. NEJM 372, 1312-23 (2015). **Additional references: 1a:** Ramanan P *et al*. Clinical Microbiology Reviews, 31 (1) e00024-17,(2017). **2a :** Amy L. Leber J Clin Microbiol, 56 : e01945-17, (2018). **3a:** Judd D *et al*. Abstract 2752, ECCMID, Spain (2018). 4a. Holter et al, PlosOne 2016.

……..

August 2019, rev. December 2019, rev. July 2020; rev. 16.09.2021

Appendix 1: Schedule of Activities	Day	Day	Day discharge
Triage based on severity scores (see below) and clinical	X		
Informed consent	X		
Inclusion and exclusion criteria	X		
Vital parameters: SaO2%, respiratory rate, MAP (mmHg), systolic and diastolic pressure, heart rate, temp, signs of confusion, need for vasopressors, Glasgow coma score, AVPU score, SATS score, TEWS (triage early warning score)	X	X	X
Demography (incl. socio-economic factors-education, no. of family members and need for assistance)	X		X (need and type of assistance on discharge)
Full physical examination including height and weight	X		
Medical history (includes substance usage and smoking* and vaccination status)<br>*smokers (current and past 5 years) will be offered spirometry 2-months post discharge to rule out an undiagnosed COPD	X		
Past and current medical conditions	X		
Concomitant medication review including PPI use, inhalers, and antibiotics (type and duration) prior	X		
Assessment of co-morbidities, including cardiovascular,	X		X
Assessment of physical function and cognitive status (handgrip test, accelerometer recordings, clinical frailty score, 4AT score)	X	X	X
Laboratory assessments			
Blood count, haemoglobin conc. urea, albumen, arterial blood gas analysis [PaO2, PaCo2, HCO3, SaO2, lactate], chemistry	X		X
Blood sampling for glycaemia- glycosylated haemoglobin A1c (HbA1c), glucose, inflammatory protein markers, 25-hydroxyvitamin D, and selected micronutrients	X		

……………………………………………………………………………………………..

Respiratory tract specimens: throat swab, nasal swab, induced sputum or tracheal suction samples. Randomization of respiratory samples will be performed at Dept. of Micro. HUS	X		
Triglycerides, lipid profiling, cholesterol, bilirubin, ASAT, ALAT, GT, ALP	X		
Creatinine (eGFR), urea, Na, K, and urine (dipsticks test). Microscopic examination of urine (if blood or protein is abnormal).	X		X
In patients with current, previous or family history of cardiovascular co-morbidity: EKG, troponin, and proBNP. In patients with h/o or suspected alcohol overuse: phosphatidylethanol in blood	X		
Blood for C-reactive protein, procalcitonin, proadrenomedullin, copeptin, white blood cell count, Lymphocyte Count Percentage, Neutrophil Count Percentage and Neutrophil/Lymphocyte Ratio	X		X
Blood in PaxGene tubes for RNA for transcriptional biomarkers	X		X

Summary Schedule of Patient Evaluations. Plasma, serum, whole blood, sputum and other respiratory samples will also be bio-banked

……………………………………………………………………………………………………..

August 2019, rev. December 2019, rev. July 2020; rev. 16.09.2021
