# Supplementary material for: Diagnostic Stewardship in Community-Acquired Pneumonia With Syndromic Molecular Testing: A Randomized Clinical Trial
Source: JAMA Netw Open. 2024 Mar 6;7(3):e240830. doi: 10.1001/jamanetworkopen.2024.0830 (PMC10918504; doi:10.1001/jamanetworkopen.2024.0830)
Supplement: Supplement 2. — eTable 1. Targets Included in the BIOFIRE FILMARRAY Pneumonia plus Panel eTable 2. Standard Comments for Detected Bacteria eTable 3. Definitions and Diagnostic Clinical Criteria for CAP eTable 4. Baseline Characteristics for CAP Patients Only (n=200) eTable 5. Pathogen-Directed Treatment Within 48 Hours for Patients With Pneumonia (n=200) eTable 6. Comparison of Length of Stay and Clinical Outcomes Between Rapid Testing by FAP Plus and Standard-of-Care for All Randomized Patients (n=374) and CAP Patients only (n=200), Respectively eTable 7. Microbiological Detections in Respiratory Samples in the Standard-of-Care and FAP Plus Arms [file jamanetwopen-e240830-s002.pdf]

Supplemental Online Content

Markussen DL, Serigstad S, Ritz C, et al. Diagnostic stewardship in community-acquired pneumonia with syndromic molecular testing: a randomized clinical trial. *JAMA Netw Open*. 2024;7(3):e240830. doi:10.1001/jamanetworkopen.2024.0830

e Table 1. Targets included in the BIOFIRE® FILMARRAY® Pneumonia plus Panel

eTable 2. Standard Comments for Detected Bacteria

eTable 3. Definitions and Diagnostic Clinical Criteria for CAP

eTable 4. Baseline Characteristics for CAP Patients Only (n=200)

eTable 5. Pathogen-Directed Treatment Within 48 Hours for Patients With Pneumonia (n=200)

eTable 6. Comparison of Length of Stay and Clinical Outcomes Between Rapid Testing by FAP Plus and Standard-of-Care for All Randomised Patients (n=374) and CAP Patients only (n=200), Respectively

eTable 7. Microbiological Detections in Respiratory Samples in the Standard-of-Care and FAP Plus Arms

This supplemental material has been provided by the authors to give readers additional information about their work.

e Table 1. Targets included in the BIOFIRE® FILMARRAY® Pneumonia *plus* Panel

Bacteria (semi quantitative)
Acinetobacter calcoaceticus-baumannii complex
Enterobacter cloacae
Escherichia coli
Haemophilus influenzae
Klebsiella aerogenes
Klebsiella oxytoca
Klebsiella pneumoniae group
Moraxella catarrhalis
Proteus spp.
Pseudomonas aeruginosa
Serratia marcescens
Staphylococcus aureus
Streptococcus agalactiae
Streptococcus pneumoniae
Streptococcus pyogenes

Atypical Bacteria (Qualitative)
Legionella pneumophila
Mycoplasma pneumoniae
Chlamydia pneumoniae

Viruses
Influenza A
Influenza B
Adenovirus
Seasonal Coronavirus
Parainfluenza virus
Respiratory Syncytial virus
Human Rhinovirus/Enterovirus
Human Metapneumovirus
Middle East Respiratory Syndrome Coronavirus (MERS-CoV)

Antibiotic Resistance Genes
ESBL
CTX-M

Carbapenemases
KPC
NDM
Oxa48-like
VIM
IMP

Methicilin Resistance
mecA/mecC and MREJ

© 2024 Markussen DL et al. *JAMA Network Open*

eTable 2. Standard comments for detected bacteria [3-20]

Respiratory tract microbes divided in different categories according to their potential clinical relevance	
**Category**	**Microbe**
**A: Always pathogens**<br>Always considered relevant in a patient with respiratory tract infection	Adenovirus [6,8,12,14]<br>Coronavirus [8,12,14]<br>Human metapneumovirus [6, 12,14]<br>Influenza virus [6,8,12,14]<br>MERS [9]<br>Parainfluenza virus [8,12,14]<br>Rhinovirus [6,12,14]<br>RS-virus [6,8,12,14]<br><br>*Bordetella pertussis* [4]<br>*Chlamydia pneumoniae* [3,6,8,12,14]<br>*Legionella pneumophila* [3,6,14]<br>*Mycoplasma pneumoniae* [3,6,8,12,14]
**B: Usually pathogens**<br>Can be colonizers, but are usually considered relevant in a patient with pneumonia	*Haemophilus influenzae* [3,6,8,12,14]<br>*Streptococcus pneumoniae* [3,6,8,12,14,20]<br>*Streptococcus pyogenes* [11,18,20]
**C: Usually not pathogens**<br>Usually colonizers, but can cause pneumonia, especially in patients with chronic diseases	*Klebsiella pneumoniae* [7,14,20]<br>*Moraxella catarrhalis* [3,5,12,14]<br>*Staphylococcus aureus* [3,12,14,15,20]
**D: Usually not pathogens**<br>A seldom cause of pneumonia in otherwise healthy patients. May be considered relevant if detected as the only bacterial species in a patient with immunosuppression and/or several previous courses of antibiotics	*Acinetobacter calcoaceticus-baumannii-complex* [4,12,20]<br>*Enterobacter cloacae* [6,12,19]<br>*Escherichia coli* [6,12,14,19,20]<br>*Klebsiella aerogenes* [6,12]<br>*Klebsiella oxytoca* [6,12]<br>*Proteus species* [6,12,19]<br>*Pseudomonas aeruginosa* [12-14,19,20]<br>*Serratia marcescens* [6,12,19]<br>*Streptococcus agalactiae* [17]

© 2024 Markussen DL et al. *JAMA Network Open*

References

3 Apisarnthanarak, A. & Mundy, L. M. Etiology of community-acquired pneumonia. *Clin Chest Med* **26**, 47-55, doi:10.1016/j.ccm.2004.10.016 (2005).

4 Asai, N. *et al.* Clinical manifestations and risk factors of community-onset Acinetobacter species pneumonia in Japan; case control study in a single institute in Japan. *J Infect Chemother* **25**, 639-642, doi:10.1016/j.jiac.2019.03.016 (2019).

5 Hirai, J. *et al.* Clinical characteristics of community-acquired pneumonia due to Moraxella catarrhalis in adults: a retrospective single-centre study. *BMC Infect Dis* **20**, 821, doi:10.1186/s12879-020-05564-9 (2020).

6 Holter, J. C. *et al.* Etiology of community-acquired pneumonia and diagnostic yields of microbiological methods: a 3-year prospective study in Norway. *BMC Infect Dis* **15**, 64, doi:10.1186/s12879-015-0803-5 (2015).

7 Lin, Y. T., Jeng, Y. Y., Chen, T. L. & Fung, C. P. Bacteremic community-acquired pneumonia due to Klebsiella pneumoniae: clinical and microbiological characteristics in Taiwan, 2001-2008. *BMC Infect Dis* **10**, 307, doi:10.1186/1471-2334-10-307 (2010).

8 Mandell, L. A. Community-acquired pneumonia: An overview. *Postgrad Med* **127**, 607-615, doi:10.1080/00325481.2015.1074030 (2015).

9 Memish, Z. A., Perlman, S., Van Kerkhove, M. D. & Zumla, A. Middle East respiratory syndrome. *Lancet* **395**, 1063-1077, doi:10.1016/S0140-6736(19)33221-0 (2020).

10 Metlay, J. P. *et al.* Diagnosis and Treatment of Adults with Community-acquired Pneumonia. An Official Clinical Practice Guideline of the American Thoracic Society and Infectious Diseases Society of America. *American Journal of Respiratory and Critical Care Medicine* **200**, e45-e67, doi:10.1164/rccm.201908-1581ST (2019).

11 Muller, M. P. *et al.* Clinical and epidemiologic features of group A streptococcal pneumonia in Ontario, Canada. *Arch Intern Med* **163**, 467-472, doi:10.1001/archinte.163.4.467 (2003).

12 Musher, D. M. & Thorner, A. R. Community-Acquired Pneumonia. *New England Journal of Medicine* **371**, 1619-1628, doi:10.1056/NEJMra1312885 (2014).

13 Restrepo, M. I. *et al.* Burden and risk factors for Pseudomonas aeruginosa community-acquired pneumonia: a multinational point prevalence study of hospitalised patients. *Eur Respir J* **52**, doi:10.1183/13993003.01190-2017 (2018).

14 Rider, A. C. & Frazee, B. W. Community-Acquired Pneumonia. *Emerg Med Clin North Am* **36**, 665-683, doi:10.1016/j.emc.2018.07.001 (2018).

15 Self, W. H. *et al.* Staphylococcus aureus Community-acquired Pneumonia: Prevalence, Clinical Characteristics, and Outcomes. *Clin Infect Dis* **63**, 300-309, doi:10.1093/cid/ciw300 (2016).

16 Skolnik, K. *et al.* Group B streptococcus (GBS) is an important pathogen in human disease-but what about in cystic fibrosis? *BMC Infect Dis* **17**, 660, doi:10.1186/s12879-017-2729-6 (2017).

17 Stralin, K., Olcen, P., Tornqvist, E. & Holmberg, H. Definite, probable, and possible bacterial aetiologies of community-acquired pneumonia at different CRB-65 scores. *Scand J Infect Dis* **42**, 426-434, doi:10.3109/00365540903552353 (2010).

18 Tamayo, E., Montes, M., Vicente, D. & Pérez-Trallero, E. Streptococcus pyogenes Pneumonia in Adults: Clinical Presentation and Molecular Characterization of Isolates 2006-2015. *PLoS One* **11**, e0152640, doi:10.1371/journal.pone.0152640 (2016).

19 von Baum, H., Welte, T., Marre, R., Suttorp, N. & Ewig, S. Community-acquired pneumonia through Enterobacteriaceae and Pseudomonas aeruginosa: Diagnosis, incidence and predictors. *Eur Respir J* **35**, 598-605, doi:10.1183/09031936.00091809 (2010).

20 Wongsurakiat, P. & Chitwarakorn, N. Severe community-acquired pneumonia in general medical wards: outcomes and impact of initial antibiotic selection. *BMC Pulm Med* **19**, 179, doi:10.1186/s12890-019-0944-1 (2019).

© 2024 Markussen DL et al. *JAMA Network Open*

eTable 3. Definitions and diagnostic clinical criteria for CAP
(Adapted from Postma D.F. et al.[1] and Serigstad S *et al* [2])

Clinical CAP:

- Patients with at least two diagnostic criteria* and in-hospital treatment and/or diagnosis of clinically suspected CAP documented by the treating physician and in agreement with the assessment by a study investigator. In case of disagreement, an additional study investigator will arbitrate.
- Patients with two or more diagnostic criteria and an obvious non-respiratory source of infection are not considered to have a clinical diagnosis of CAP.

Radiologically confirmed CAP:

- Clinical CAP
- The presence of a new or increased infiltrate on chest radiography or computer tomography (CT).

*** Diagnostic criteria:**

- Cough: recent or worsening
- Production of purulent sputum or a change in the character of sputum
- Temperature >38.0°C or <36.1°C
- Auscultatory findings consistent with pneumonia, including rales, evidence of pulmonary consolidation (dullness on percussion, bronchial breath sounds, or egophony), or both
- Leukocytosis (>11.0 × 10^9 white cells per liter or > 8.2 x 10^9 neutrophils)
- C-reactive protein (CRP) level > 50 mg/L
- Dyspnea, tachypnea, or hypoxemia: recent or worsening

References

1 Postma, D. F. *et al.* Antibiotic treatment strategies for community-acquired pneumonia in adults. *N Engl J Med* **372**, 1312-1323, doi:10.1056/NEJMoa1406330 (2015).

2. Rapid syndromic PCR testing in patients with respiratory tract infections reduces time to results and improves microbial yield. Serigstad S, Markussen D, Grewal HMS, Ebbesen M, Kommedal Ø, Heggelund L, van Werkhoven CH, Faurholt-Jepsen D, Clark TW, Ritz C, Ulvestad E, Bjørneklett R, Knoop ST; CAPNOR Study Group. Sci Rep. 2022 Jan 10;12(1):326. doi: 10.1038/s41598-021-03741-7.

eTable 4. Baseline characteristics for CAP patients only (n=200)

	**FAP *plus*** (n=97)	**Standard-of-care** (n=103)
Age (years)	71 (60, 82)	72 (62, 80)
Sex (% Female)	40 (39/97)	42 (43/103)
Current smoker	21 (20/97)	13 (14/103)
Influenza vaccine	53 (52/97)	58 (60/103)
Pneumococcal vaccine	38 (37/97)	37 (38/103)
Duration of symptoms (days)	6.5 (3.6, 8.7)	5.8 (3.5, 7.6)
Antibiotics with 48 hours	23 (22/97)	17 (18/103)
Antibiotics within the last month (%)	15 (15/97)	9 (9/103)
Comorbidities		
Hypertension	37 (36/97)	39 (40/103)
Cardiovascular disease	34 (33/97)	30 (31/103)
Respiratory disease	54 (52/97)	50 (51/103)
Renal disease	11 (11/97)	9 (9/103)
Liver disease	0 (0/97)	2 (2/103)
Diabetes	8 (8/97)	14 (14/103)
Immunocompromised	8 (8/97)	12 (12/103)
Cancer	9 (9/97)	7 (7/103)
Charlson Comorbidity Index score	4 (2, 5)	4 (2, 5)
Observations		
Temperature (°C)	37.2 (36.8, 37.6)	37.2 (36.8, 37.7)
Pulse rate (bpm)	92 (78, 109)	94 (85, 110)

© 2024 Markussen DL et al. *JAMA Network Open*

Respiratory rate (bpm)	22 (20, 25)	24 (20, 28)
O_2 saturations (%)	93 (90, 96)	93 (89, 96)
Supplementary O_2	6 (6/97)	6 (6/103)
Blood pressure (mmHg)		
Systolic	127 (116, 146)	134 (117, 148)
Diastolic	80 (71, 86)	80 (70, 88)
Laboratory and radiology		
CRP (mg/L)	135 (83, 209)	151 (101, 237)
White blood cell count ($\times 10^9$ per L)	11.3 (8.1, 16.7)	11.9 (8.7, 15.0)
Chest X-ray (%)	96 (93/97)	97 (100/103)
Chest CT (%)	24 (23/97)	20 (21/103)
CRB-65	1 (1, 2)	1 (1, 2)
qSOFA	1 (0, 1)	1 (0, 1)
SARS-CoV-2 positive test	15 (15/97)	16 (16/103)

Data are shown as percentage (count) and median (IQR) for binary and continuous variables, respectively.

IQR = interquartile range. O_2=oxygen. CRP = C-reactive protein. CT = computed tomography. CRP and white blood cell counts presented are the highest values during hospitalization. *CRB-65=* confusion, respiratory rate, blood pressure, and age > 65 years; qSOFA= quick sequential organ failure assessment score.

© 2024 Markussen DL et al. *JAMA Network Open*

eTable 5. Pathogen-directed treatment within 48 hours for patients with pneumonia (n=200)

Pathogen-directed treatment	Test that initiated pathogen-directed treatment	Standard of care N=16		FAP *plus* N=46	
		N	Pathogen-directed antibacterial therapy	N	Pathogen-directed antibacterial therapy
**Continuation of appropriate empirical treatment**	Blood cultures	0		2	PenG: 2
	LRT culture	1	PenG: 1	0	
	FAP *plus*	0		13	PenG: 11<br>Carbapenems: 1<br>Pip/Tazo: 1
	PUAT	6	PenG: 5<br>Ampicillin: 1	1	PenG: 1
	Total	7		16	
**Escalation to more broad-spectrum treatment**	Blood cultures	3	PenG to 3GC: 2<br>PenG to a more broad-spectrum penicillin: 1	0	
	LRT culture	1	PenG to a more broad-spectrum penicillin: 1	0	
	FAP *plus*	0		14	PenG changed to a more broad-spectrum penicillin: 6<br>Benzylpenicillin to 3GC: 5<br>Aminoglycoside added: 2<br>Other: 1
	Total	4		14	
**De-escalation to more narrow-spectrum treatment**	Other	1*	3GC to quinolone	0	
	LRT culture	2		0	
	FAP *plus*	0		10	Stopped aminoglycosides: 5<br>Stopped macrolides: 3<br>Stopped clindamycin: 1<br>3GC to ciprofloxacin: 1
	PUAT	4	Stopped aminoglycosides: 3<br>3GC to PenG: 1	0	
	Total	5		10	
**Initiated pathogen-directed antimicrobial treatment (without prior empirical antibiotic treatment)**	FAP *plus*	0		6	PenG: 2<br>Ampicillin: 1<br>Amoxi/Clav:1<br>3GC: 2
	Total	0		6	

Abbreviations: FAP *plus* - Biofire® Filmarray® Pneumonia plus Panel; LRT culture – lower respiratory tract sample culture; PUAT – pneumococcal urine antigen test; PenG – Benzylpenicillin; 3GC - third generation cephalosporins; Pip/Tazo - Piperacillin/Tazobactam; TMP/S - Trimethoprim/sulfamethoxazole; Amoxi/Clav - Amoxicillin/Clavulanic acid

*) *Legionella pneumohilia* PCR

eTable 6. Comparison of length of stay and clinical outcomes between rapid testing by FAP *plus* and standard-of-care for all randomised patients (n=374) and CAP patients only (n=200), respectively

	**FAP *plus***	**Standard-of-care**	**FAP *plus* vs. Standard-of-care#**		
***All patients** (n=374)*			**Difference**	**Odds ratio**	**p-value**
Readmission	15.5 (29/187)	18.7 (35/187)	-3.2 (-10.8, 4.4)	0.80 (0.46, 1.37)	0.41
30-day mortality	4.8 (9/187)	3.7 (7/187)	1.1 (-3.0, 5.2)	1.30 (0.47, 3.71)	0.61
90-day mortality	8.6 (16/187)	5.9 (11/187)	2.7 (-2.6, 7.9)	1.50 (0.68, 3.41)	0.32
				**Ratio of medians**	
Length of stay (days)	3.3 (2.0, 6.0)	3.2 (2.0, 6.0)	0.15 (-0.55, 0.85)	1.05 (0.83, 1.34)	0.67
***CAP patients** (n=200)*				**Odds ratio**	
Readmission	14.4 (14/97)	18.4 (19/103)	-4.0 (-14.3, 6.2)	0.75 (0.35, 1.58)	0.45
30-day mortality	3.1 (3/97)	3.9 (4/103)	-0.8 (-5.9, 4.3)	0.79 (0.15, 3.67)	0.76
90-day mortality	7.2 (7/97)	5.8 (6/103)	1.4 (-5.5, 8.2)	1.26 (0.40, 4.04)	0.69
				**Ratio of medians**	
Length of stay (days)	3.9 (2.9, 6.1)	4.0 (2.2, 6.3)	0.48 (-0.48, 1.45)	1.15 (0.87, 1.51)	0.33

Data shown as percentage (count) for binary outcomes and as median (IQR) for length of stay.

#: Differences were estimated as risk differences for binary outcomes (on readmission and mortality) and differences of median for length of stay using unadjusted logistic regression and linear regression with logarithm-transformed length of stay, respectively. Odds ratios for binary outcomes and ratio of medians for length of stay were estimated using unadjusted logistic and linear regression with logarithm-transformed length of stay, respectively. p-values corresponding to testing ratios equal to 1 were reported.

FAP *plus,* BioFire FilmArray Pneumonia panel *plus* (bioMérieux S.A., Marcy-l'Etoile, France); CAP, community-acquired pneumonia; IQR, interquartile range.

eTable 7. **Microbiological detections in respiratory samples in the standard-of-care and FAP *plus* arms**

**Microbe detected**	**All randomized patients**		**Patients with pneumonia and provided an LRT sample**	
	Standard of care (n=187)	FAP plus (n=187)	Standard of care (n=103)	FAP plus (n=97)
Acinetobacter calcoaceticus-baumannii complex	0	4	0	1
Enterobacter cloacae complex	0	4	0	4
Escherichia coli	5	13	3	5
Haemophilus influenzae	22	40	18	29
Klebsiella aerogenes	0	0	0	0
Klebsiella oxytoca	1	6	1	3
Klebsiella pneumoniae group	2	5	1	3
Moraxella catarrhalis	11	16	9	11
Proteus spp.	0	3	0	1
Pseudomonas aeruginosa	5	10	4	4
Serratia marcescens	2	2	0	1
Staphylococcus aureus	7	36	4	23
Streptococcus agalactiae	0	9	0	6
Streptococcus pneumoniae	14	26	14	21
Streptococcus pyogenes	0	1	0	1
Legionella pneumophila	1	0	1	0
Mycoplasma pneumoniae	0	0	0	0
Chlamydia pneumoniae	0	0	0	0
Other bacterial pathogens not included in the FAP *plus* panel*	3	0	2	0
**Total number of bacterial detections**	**72**	**175**	**57**	**113**
SARS-CoV-2**	24	28	16	15
Human rhinovirus/enterovirus	14	22	5	12
Parainfluenza virus	7	4	2	0
Respiratory syncytial virus	10	15	4	7
Seasonal coronavirus***	0	7	0	5
Human metapneumovirus	7	0	5	0
Adenovirus	0	1	0	0
Influenza A virus	1	0	1	0
Influenza B virus	1	0	1	0
**Total number of viral detections**	**63**	**74**	**34**	**39**

Abbreviations: FAP *plus,* Biofire FilmArray Pneumonia panel *plus*; SARS-CoV-2, severe acute respiratory syndrome coronavirus 2.

*) One detection of *S. dysgalactiae* and two detections of *K. variicola*

© 2024 Markussen DL et al. *JAMA Network Open*

**) SARS-CoV-2 is not a target in the FAP *plus* and was tested using laboratory PCR.
***) Seasonal human coronavirus includes: HKU1, 229E, NL63 and OC43. Testing for seasonal coronavirus was not part of the standard of care diagnostics until May 2022.

© 2024 Markussen DL et al. *JAMA Network Open*
