# Supplementary material for: Diagnostic Stewardship in Community-Acquired Pneumonia With Syndromic Molecular Testing: A Randomized Clinical Trial
Source: JAMA Netw Open. 2024 Mar 6;7(3):e240830. doi: 10.1001/jamanetworkopen.2024.0830 (PMC10918504; doi:10.1001/jamanetworkopen.2024.0830)
Supplement: Supplement 3. — Data Sharing Statement [file jamanetwopen-e240830-s003.pdf]

Data Sharing Statement

Markussen. Diagnostic Stewardship in Community-Acquired Pneumonia With Syndromic Molecular Testing. *JAMA Netw Open*. Published March 06, 2024.
doi:10.1001/jamanetworkopen.2024.0830

Data
Data available: Yes
Data types: Deidentified participant data, Data dictionary
How to access data: All de-identified participant data analysed and presented in this study are available from the corresponding author following publication upon reasonable request.
When available: With publication

Supporting Documents
Document types: Other (please specify)
Additional Information: All de-identified participant data analysed and presented in this study are available from the corresponding author following publication upon reasonable request.
How to access documents: All de-identified participant data analysed and presented in this study are available from the corresponding author following publication upon reasonable request.
When available: With publication

Additional Information
Who can access the data: Data sharing will be permitted with researchers whose proposed use of the data has been approved.
Types of analyses: Data sharing requests within three years of publication should be made to the first author at Harleen.Grewal@UiB.no for the provision of the data dictionary and data request form.
Mechanisms of data availability: with a signed data access agreement
